# Osimertinib in combination with anti-angiogenesis therapy presents a promising option for osimertinib-resistant non-small cell lung cancer

**DOI:** 10.1186/s12916-024-03389-w

**Published:** 2024-04-24

**Authors:** Ruoshuang Han, Haoyue Guo, Jinpeng Shi, Sha Zhao, Yijun Jia, Xiaozhen Liu, Yiwei Liu, Lei Cheng, Chao Zhao, Xuefei Li, Caicun Zhou

**Affiliations:** 1 Department of Medical Oncology, Shanghai Pulmonary Hospital, Tongji University School of Medicine, Shanghai, People’s Republic of China; 2 Department of Oncology, The First Affiliated Hospital of Army Medical University, Chongqing, People’s Republic of China; 3 Department of Lung Cancer and Immunology, Shanghai Pulmonary Hospital, Tongji University School of Medicine, Shanghai, People’s Republic of China

**Keywords:** Osimertinib, Drug resistance, Anti-angiogenesis, Tumor microenvironment

## Abstract

**Background:**

Osimertinib has become standard care for epidermal growth factor receptor (EGFR)-positive non-small cell lung cancer (NSCLC) patients whereas drug resistance remains inevitable. Now we recognize that the interactions between the tumor and the tumor microenvironment (TME) also account for drug resistance. Therefore, we provide a new sight into post-osimertinib management, focusing on the alteration of TME.

**Methods:**

We conducted a retrospective study on the prognosis of different treatments after osimertinib resistance. Next, we carried out in vivo experiment to validate our findings using a humanized mouse model. Furthermore, we performed single-cell transcriptome sequencing (scRNA-seq) of tumor tissue from the above treatment groups to explore the mechanisms of TME changes.

**Results:**

Totally 111 advanced NSCLC patients have been enrolled in the retrospective study. The median PFS was 9.84 months (95% CI 7.0–12.6 months) in the osimertinib plus anti-angiogenesis group, significantly longer than chemotherapy (*P* = 0.012) and osimertinib (*P* = 0.003). The median OS was 16.79 months (95% CI 14.97–18.61 months) in the osimertinib plus anti-angiogenesis group, significantly better than chemotherapy (*P* = 0.026), the chemotherapy plus osimertinib (*P* = 0.021), and the chemotherapy plus immunotherapy (*P* = 0.006).

The efficacy of osimertinib plus anlotinib in the osimertinib-resistant engraft tumors (R-O+A) group was significantly more potent than the osimertinib (R-O) group (*P*<0.05) in vitro. The combinational therapy could significantly increase the infiltration of CD4^+^ T cells (*P<*0.05), CD25^+^CD4^+^ T cells (*P*<0.001), and PD-1^+^CD8^+^ T cells (*P<*0.05) compared to osimertinib.

ScRNA-seq demonstrated that the number of CD8^+^ T and proliferation T cells increased, and TAM.mo was downregulated in the R-O+A group compared to the R-O group. Subtype study of T cells explained that the changes caused by combination treatment were mainly related to cytotoxic T cells. Subtype study of macrophages showed that proportion and functional changes in IL-1β.mo and CCL18.mo might be responsible for rescue osimertinib resistance by combination therapy.

**Conclusions:**

In conclusion, osimertinib plus anlotinib could improve the prognosis of patients with a progressed disease on second-line osimertinib treatment, which may ascribe to increased T cell infiltration and TAM remodeling via VEGF-VEGFR blockage.

**Supplementary Information:**

The online version contains supplementary material available at 10.1186/s12916-024-03389-w.

## Background

Lung cancer mortality is the highest among all cancers [1]. In the past decade, non-small cell lung cancer (NSCLC) patients with epidermal growth factor receptor (EGFR) mutations, such as exon 19 deletions and exon 21 L858R point mutations, have benefited from EGFR tyrosine kinase inhibitors (TKIs) [2, 3]. Based on the AURA research, the third-generation EGFR-TKI osimertinib (AZD9291) has become standard care for T790M-positive NSCLC patients who experience progression after EGFR-TKI [4]. However, most patients undergoing osimertinib treatment group eventually develop resistance to EGFR-dependent or-independent mechanisms. The C797S mutation, a missense mutation in the exon 20 tyrosine kinase region of the EGFR gene that prevents covalent binding of osimertinib to the ATP site, is the most common tertiary EGFR mutation that mediates osimertinib resistance [5]. NGS testing of plasma specimens from 73 patients before and after resistance in the AURA3 study revealed that 14% developed the C797S mutation after second-line osimertinib treatment. Other tertiary mutations included L792X, G796S, L718Q, and S768I. In addition to tertiary mutations in EGFR, EGFR gene amplification is also a cause of acquired resistance to osimertinib [6]. Alternative bypass activation, represented by c-MET amplification, can activate the Ras-MAPK and PI3K-Akt downstream pathways and mediate osimertinib resistance, which occurs in approximately 19% (second-line treatment) of patients after osimertinib resistance [7]. Other common bypass activations that lead to resistance to osimertinib are HER2 amplification, FGFR, IFG1R, and abnormal activation of the AXL pathways [8]. In addition to bypass activation, the activation of EGFR downstream pathways due to BRAF mutations, KRAS mutations, and PIK3CA amplification can also lead to resistance [9]. Tissue phenotype switching is also a major cause of resistance, with an incidence of tissue phenotype switching of 14% in studies of osimertinib for second-line therapy [10].

Based on these extensive resistance mechanism studies, solutions to overcome osimertinib resistance can be broadly classified in two ways. They are exploring a new generation of EGFR-TKIs for EGFR-dependent resistance and developing combination therapies for EGFR-independent resistance [11]. Treatment resistance due to the EGFR C797S mutation is determined by the relative positions of the C797S and T790M mutations. Treatment with osimertinib in combination with specific target inhibitors is mainly used for drug resistance owing to bypass or downstream pathway activation. Platinum-based chemotherapy is mainly used for resistance caused by the phenotypic transformation [12].

The current NCCN clinical guidelines for the treatment of osimertinib resistance are mainly based on clinical symptoms and disease progression patterns. The main post-osimertinib treatment strategies in clinical settings are: when the disease is asymptomatic and slowly progressing, treatment with osimertinib can be continued; when oligometastases appear, local ablation therapy (LAT) or stereotactic radiotherapy (SBRT) can be added; when rapid and systemic progression occurs, another systemic therapy, mainly cytotoxic chemotherapy, is required; therapy based on resistance mechanisms revealed by NGS testing of post-resistance specimens is also recommended [10]. With the exhaustion of targeted therapy options, several clinical studies have explored whether combining targeted therapy with other therapies after osimertinib resistance could improve the comprehensive effects [13]. A retrospective study showed that osimertinib-based combination had better overall survival (OS) than cytotoxic chemotherapy (not achieved vs.7.8 months; 95% CI 0.17–0.89, *P*<0.05) [14]. However, studies have shown that the median duration of treatment (mDOT) of osimertinib combined with chemotherapy was not significantly longer than that of chemotherapy alone [15, 16]. In the phase Ib TATTON study, osimertinib combined with the PD-L1 inhibitor durvalumab was discontinued because of serious adverse effects, such as interstitial pneumonia (38%). The phase II ALTER-L031 study aims to evaluate the efficacy of the vascular endothelial growth factor receptor (VEGFR) inhibitor anlotinib combined with chemotherapy [10]. Furthermore, the efficacy of anlotinib in combination with chemotherapy and another PD-1 inhibitor, toripalimab, is also being assessed after osimertinib failure [10]. Research on the validity of different post-osimertinib treatments is required. We present a long-term and real-world retrospective study that analyzed the survival data of post-osimertinib therapies, hoping to provide clues for managing osimertinib resistance.

Previous discussions on drug resistance in NSCLC treatment mainly focused on the intrinsic mechanisms of tumor cells. Currently, growing evidence indicates that their behavior is also affected by the tumor environment in which they grow. Efforts to unravel the features of cancer ecosystems will drive the development of more nuanced approaches [17]. The tumor microenvironment (TME) comprises heterogeneous components such as cancer cells, immune cells, stromal cells, endothelial cells, cytokines, and chemokines [18]. We now recognize that the interactions between tumor cells and the TME are heterogeneous and dynamic, and mutual domestication accounts for drug resistance and tumor metastasis [19, 20]. From this perspective, future NSCLC treatment should be based on genotype and consider TME remodeling.

Based on the ever-accumulating evidence that the communication between the tumor and TME determines patient fate, it is vital to explore TME changes after osimertinib changes, which cannot be achieved without suitable mouse models. The mouse models currently used for immune microenvironment studies are divided into immunocompetent mouse homograft tumor models and humanized mouse models. Transplanting murine-derived tumor cells (e.g., Lewis lung cancer cells) into immunocompetent mice (e.g., C57BL/6 mice) is currently the most common model in tumor immunity research [21]. The most significant drawback of this allograft model is that the tumor biology and microenvironment of mice are different from those of humans, which may impede the transfer of results to the clinic [22]. Moreover, there are no appropriate murine cell lines that we need in some specific studies. Therefore, some studies have adopted the “humanized” model of transplanting human-derived tumor cells in immunocompetent mice [23]. The most commonly used mouse for humanized models are the severe combined immunodeficient NOG (NOD-Prkdc^scid^Il2rg^nul^ ) mice, with a non-obese diabetic mouse NOD genetic background, SCID gene mutations, and knockout of the gamma chain subunit of the IL-2 receptor [24]. Humanized mouse models can be further subdivided into several categories depending on the source of tumor cells (cell-derived xenograft (CDX) or patient-derived xenograft (PDX)) and human immune cells (peripheral blood mononuclear cells (PBMCs) or hematopoietic stem cells (HSCs)) [25, 26].

In this study, we conducted a real-world retrospective study on the prognosis of different treatments after second-line osimertinib resistance. Next, we carried out an in vivo experiment to validate our findings in the above retrospective research and explore TME changes upon osimertinib treatment using a humanized mouse model. Furthermore, we performed single-cell transcriptome sequencing of the fresh transplanted tumor tissue to analyze the components, functions of cell subpopulations in the TME, and their mutual communication to explore the potential mechanisms of TME changes. Our study provides insight into treatment after osimertinib in the real world and reveals the potential corresponding TME changes related to osimertinib resistance.

## Methods

### Patients

We retrospectively evaluated a cohort of 492 NSCLC patients harboring *EGFR* T790M mutations between January 2016 and May 2019 at the Shanghai Pulmonary Hospital. The inclusion criteria were as follows: (1) age over 18 years; (2) histologically or cytologically confirmed diagnosis of lung adenocarcinoma or NSCLC not otherwise specified (NSCLC-NOS); (3) stage IIIB/IV based on the 8th AJCC TNM staging guide [27]; (4) sensitive *EGFR* mutations including in-frame deletion in exon 19 (19del) and L858R point mutation in exon 21 detected at baseline; (5) acquired *EGFR* T790M mutation detected when resistant to the first-generation EGFR-TKI treatment as first-line regimen; (6) osimertinib, a third-generation EGFR-TKI, received as second-line targeted therapy; (7) at least two cycles of systemic chemotherapy or immunotherapy after line beyond osimertinib resistance; (8) measurable lesion; and (9) complete case information for further study. The exclusion criteria were as follows: (1) *EGFR* T790M detected at baseline; and (2) missing data on clinicopathological characteristics and follow-up.

### Data collection

Clinicopathological characteristics were collected on the diagnosis. Age, sex, Eastern Cooperative Oncology Group performance status (ECOG PS), smoking history, source of the biopsy specimen, histology, type of mutation, metastasis, TNM stage, progression pattern on osimertinib, re-biopsy after osimertinib progression, mechanism of osimertinib resistance, local consolidative therapy (LCT), systemic treatments, and clinical outcomes of the enrolled patients were extracted from the electronic medical records. Patients who had smoked fewer than 100 cigarettes during their lifetime were never smokers.

Radiological imaging series representing at least one measurable lesion performed before and during treatment were reviewed for each patient. Chest computed tomography scans were obtained after the first month of treatment and every 4–8 weeks as a routine clinical procedure to assess efficacy. Other imaging examinations (e.g., liver ultrasound, bone emission computed tomography, and brain magnetic resonance imaging) were performed when necessary.

Tumor response was evaluated according to RECIST 1.1 [28] and recorded as complete response (CR), partial response (PR), stable disease (SD), or progressive disease (PD). The objective response rate (ORR) was reported as the proportion of patients with CR or PR, and the disease control rate (DCR) was defined as the proportion of patients with an objective response or SD (for at least 6 weeks). The median progression-free survival (mPFS) was calculated as the duration from the first day of treatment beyond osimertinib progression (third-line treatment) until disease progression or the date of death (from any cause). The median overall survival (mOS) was calculated from the first day of treatment beyond osimertinib progression (third-line treatment) to death. The last follow-up date was November 11, 2021. The RECIST efficacy assessment was conducted by experienced oncologists who were trained in interpreting radiological images and familiar with RECIST criteria guidelines. They independently evaluated the imaging data to assess treatment response. Therapy decisions were made by professional oncologist in our hospital.

### Establishment of humanized immunodeficiency mice

In our study, we adopted the huHSC-NOG-EXL-CDX model to achieve complete reconstruction of the immune system with high consistency, with a long observation window period without graft versus host diseases (GVHDs). Six- to eight-week-old female humanized NOG-EXL mice (NOG-hGM-CSF/hIL-3) were exposed to X-ray irradiation at a dose of 2 Gy and engrafted with human CD34^+^ hematopoietic stem cells (Charles River Laboratories) through the tail vein. Human immune cell reconstitution was checked by flow cytometry at 8–11 weeks post-engraftment, and the CD45^+^ engraftment rate (hCD45^+^/mCD45^+^) should be more than 25%.

### Construction of mice xenograft models

We subcutaneously injected 6 × 10^6^ H1975 (EGFR ^L858R/T790M^) or H1975-osimertinib-resistant (OR) cells (established by stepwise dose escalation) in 150 μl of serum-free culture medium into the right flanks of the above-humanized mice. Eight mice were divided equally into four groups when the tumor size reached approximately 120–180 mm^3^, mice allocated in each group were counterbalanced by weight and tumor volume. The treatment groups were as follows: (a) H1975 tumors treated with osimertinib (S-O group); (b) H1975-OR tumors treated with osimertinib (R-O group); (c) H1975-OR tumors treated with anlotinib (R-A group); and (d) H1975-OR tumors treated with osimertinib and anlotinib (R-O+A group). Blinding procedure was implemented to reduce the potential bias in interpreting the results, ZS and JY were aware of the group allocation at the different stages of experiment.

Osimertinib was administered at a dose of 5 mg/kg daily via oral gavage, and anlotinib was administered at a dose of 1 mg/kg daily via oral gavage. Mouse tumor volume and weight were measured once every 3 days. The tumor volume was calculated using the following equation: volume = length × width^2^ / 2. Tumor growth and mouse weight curves were used to evaluate the therapeutic efficiency and toxicity. After a 28-day-long treatment, the mice were euthanized. The mice were anesthetized by intraperitoneal injection of pentobarbital sodium, and euthanized by neck dissection. Tumor tissues and blood samples were collected from cardiac punctures for future analysis. We conducted animal experiments in accordance with ARRIVA guidelines.

### Flow cytometry

Fresh tumor tissues were processed into single-cell suspensions using a gentleMACS™ Dissociator with a Human Tumor Dissociation Kit (Miltenyi Biotec). Two flow cytometry panels focused on T cells and myeloid cells were applied to single-cell suspensions of blood and tumors. All cell suspensions were initially incubated with a Leukocyte Activation Kit (BD Pharmingen) at 37 °C for 5 h and then divided into two tubes (1 × 10^6^ cells/tube). Each tube was stained for viability and blocked with the Fc-block reagent (BD Pharmingen). The cells were then stained for surface markers (Additional file 1: Table S1-2). After fixation and permeabilization, cells were stained for intracellular markers (Additional file 1: Table S1-2). The limits for quadrant markers were always set based on negative populations. Cells were acquired using a Cytek Aurora cytometer and analyzed using FlowJo 10.8.0 software.

### Single-cell sequencing

Tumor-infiltrating immune cells were enriched with CD45^+^ microbeads (Miltenyi Biotech). The enriched CD45^+^ immune cells and the remaining CD45^−^ cells from each sample were mixed at a ratio of 9:1. The samples in each treatment group were mixed at a 1:1 ratio for further library construction and sequencing. We further conducted library construction and transcriptional sequencing using the Single-Cell Analysis System (BD Rhapsody) with standard manufacturing instructions.

Cell Ranger converts single-cell data from FASTQ files into cell expression matrices. Batch differences were removed based on the number of genes expressed in single cells (nFeature_RNA) and the percentage of mitochondrial mRNA (percent. mt), and percentage of ribosomes (percent. ribo). The primary screening criteria were as follows: a minimum of 200 genes were detected in each cell, and each gene was expressed in at least ten cells. The filtering criteria were as follows: (a) 200<nFeature_RNA<5000; (b) percentage. mt < 20, and (c) percent. ribo <35.

The Seurat package uses a uniform manifold approximation and a projection (UMAP) algorithm for dimension reduction. The Wilcoxon algorithm was used to identify marker genes of cell clusters, which were scored as group one vs. rest. Genes with high specific expression in each cluster, logFC>0.25, and expressed in at least 20% of the cluster were selected as significant marker genes. Furthermore, cells were annotated into major types using SingleR, using GRCh38 as a reference library. The differentially expressed genes (DEGs) among cell subsets were identified based on avg_logFC>0.2 and pct.1 or pct.2≥0.1, with which Gene Ontology (GO) and Kyoto Encyclopedia of Genes and Genomes (KEGG) enrichment analyses were conducted. Moreover, we identified cell-cell interactions by mapping receptor-ligand pairs onto cell subsets using the Cell Phone DB database [29].

### Statistical analysis

Preclinical experiment data were plotted by GraphPad Prism 8.0. One-way ANOVA were used to analyze variances, as appropriate. Statistical significance levels are indicated as *, *P* < 0.05; **, *P* < 0.01; ***, *P* < 0.001; ns, not significant. Response to treatment was regarded as a categorical variable using descriptive statistics analyzed using the Pearson *χ*^2^ test or Fisher exact test. Survival analysis was performed using the Kaplan-Meier method, and significant differences between subgroups were compared using the log-rank test. Cox regression analysis was used to test the correlation between clinicopathological characteristics and survival. HR with 95% CI was calculated, and statistical significance was set at *P* < 0.05 (two-sided) was considered statistically significant. All statistical analyses were conducted using SPSS 22.0.

## Results

### Basic characteristics of enrolled patients

**Fig. 1:**
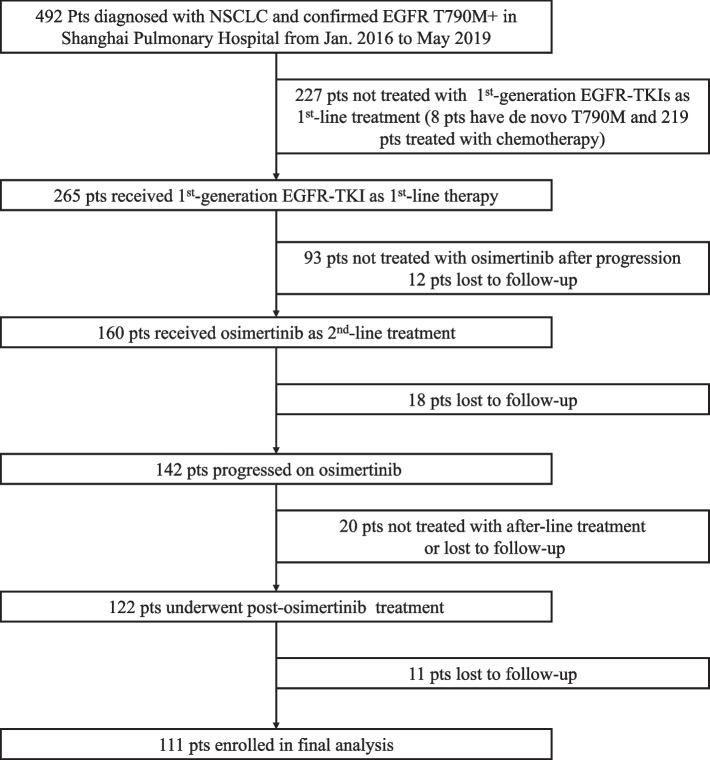
Flow diagram of patient enrollment

One hundred and eleven patients with advanced NSCLC who met the inclusion criteria were enrolled in this study. A flowchart of the patient inclusion/exclusion process is shown in Fig. 1. The clinicopathological characteristics of the enrolled patients in the different treatment groups are shown in Table 1.

**Table 1:** Characteristics of enrolled patients in different treatment groups (*N* = 111)

Variables		Total num	Chemo (*n*=45)	Chemo+Anti-angio (*n*=14)	Chemo+OSI (*n*=5)	Chemo+ICB (*n*=8)	OSI (*n*=24)	OSI+Anti-angio (*n*=11)	BSC (*n*=4)	*P*
Gender	Male	52	24	8	3	3	9	3	2	0.61
	Female	59	21	6	2	5	15	8	2	
Age	<65 y.o.	72	32	11	3	5	9	9	3	0.08
	≥65 y.o.	39	13	3	2	3	15	2	1	
ECOG PS	0–1	91	35	12	3	7	21	10	3	0.72
	2–3	20	10	2	2	1	3	1	1	
Smoking history	Non-smoke	88	34	11	4	8	19	9	3	0.86
	Smoke	23	11	3	1	0	5	2	1	
Histology	Adenocarcinoma	100	38	13	5	8	23	10	3	0.54
	NSCLC-NOS	11	7	1	0	0	1	1	1	
TMN stage	IIIB	9	5	0	0	0	1	2	1	0.37
	IV	102	40	14	5	8	23	9	3	
Biopsy specimens	Pulmonary tissue	47	21	4	0	5	9	6	2	0.01
	Blood	42	19	3	3	1	13	1	2	
	Hydrothorax	14	2	6	0	0	2	4	0	
	Lymph node	6	2	1	1	2	0	0	0	
	Unknown	2	1	0	1	0	0	0	0	
Mutation type	19Del+T790M	71	26	12	3	6	16	6	2	0.56
	L858R+T790M	40	19	2	2	2	8	5	2	
Metastasis before osimertinib treatment	No	8	2	0	0	0	3	2	1	0.22
	Intrapulmonary metastasis	21	6	5	1	1	5	3	0	
	1–3 Extrapulmonary metastasis	47	24	7	1	4	7	4	0	
	>3 Extrapulmonary metastasis	35	13	2	3	3	9	2	3	
Progression patterns	Asymptomatic	26	9	5	0	1	8	2	1	0.38
	Brain metastasis	4	2	0	1	1	0	0	0	
	Systemic limited metastasis	28	10	1	1	3	10	2	1	
	Systemic multiple lesions	12	5	1	0	0	3	2	1	
	Unknown	41	19	7	3	3	3	5	1	
Re-biopsy	No	70	30	9	5	3	14	7	2	0.43
	Yes	41	15	5	0	5	10	4	2	
Resistance mechanism of osimertinib	On-target mutation	11	4	1	0	0	4	2	0	0.37
	Off-target mutation	3	0	1	0	1	1	0	0	
	SCLC transformation	3	2	0	0	0	0	0	1	
	Unknown	94	39	12	5	7	19	9	3	
LCT	Yes	20	8	1	1	2	7	1	0	0.56
	No	91	37	13	4	6	17	10	4	

*y.o.* Years old, *ECOG PS* Eastern Cooperative Oncology Group performance status, *Num* Number, *NSCLC-NOS* NSCLC not otherwise specified, *LCT* Local consolidative therapy, *Chemo* Chemotherapy, *Anti-angio* Anti-angiogenesis therapy, *OSI* osimertinib; *ICB* Immunotherapy, *BSC* Best support care

Post-osimertinib treatment, referring to subsequent treatment after osimertinib failure, were classified as following patterns: chemotherapy (*n* = 45, 40.5%), chemotherapy plus anti-angiogenic therapy (*n* = 14, 12.6%), chemotherapy plus osimertinib (*n* = 5, 4.5%), chemotherapy plus immunotherapy (*n* = 8, 7.2%), osimertinib monotherapy (*n* = 24, 21.6%), osimertinib plus anti-angiogenic therapy (*n* = 11, 9.9%), and best supportive care (*n* = 4, 3.6%). LCT was administered to 20 patients (18.0%) in this cohort. Among the baseline clinicopathological characteristics, a statistical difference was observed only in the source of the biopsy specimens (*P* = 0.01) between the different treatment groups. No statistical differences were found according to the other characteristics in the different treatment groups.

### Osimertinib plus anti-angiogenesis therapy demonstrated better prognosis after osimertinib resistance

A summary of the best responses to diverse treatment patterns following osimertinib resistance is presented in Table 2. Regardless of treatment pattern, PR, SD, and PD were the best responses in 30 (27.0%), 66 (59.5%), and 15 (13.5%) patients, respectively. The calculated ORR was 27.0% within the entire cohort and the calculated DCR was 86.5%. Statistically significant differences were found among the different treatment groups in the ORR (*P* < 0.01) and DCR (*P* < 0.01). In particular, the group with the best ORR was chemotherapy plus osimertinib (60.0%), and the DCR of chemotherapy plus either osimertinib or immunotherapy (100.0%) was superior to that of other treatment patterns.

**Table 2:** Summary of the best responses to different post-osimertinib treatments

Variables		Chemotherapy	Chemotherapy+Anti-angiogenesis	Chemotherapy+Osimertinib	Chemotherapy+Immunotherapy	Osimertinib	Osimertinib+Anti-angiogenesis	Best support care	*P*
CR	-	-	-	-	-	-	-	-	
PR	30	12	2	3	2	5	6	0	
SD	66	26	11	2	6	15	4	2	
PD	15	7	1	0	0	4	1	2	
ORR(%)	27.0	26.7	14.3	60.0	25.0	20.8	54.5	0.0	<0.01
DCR(%)	86.5	84.4	92.9	100.0	100.0	83.3	90.9	50.0	<0.01

*CR* Complete response, *PR* Partial response, *SD* Stable disease, *PD* Progressive disease,*ORR* Objective response rate, *DCR* Disease control rate

**Fig. 2:**
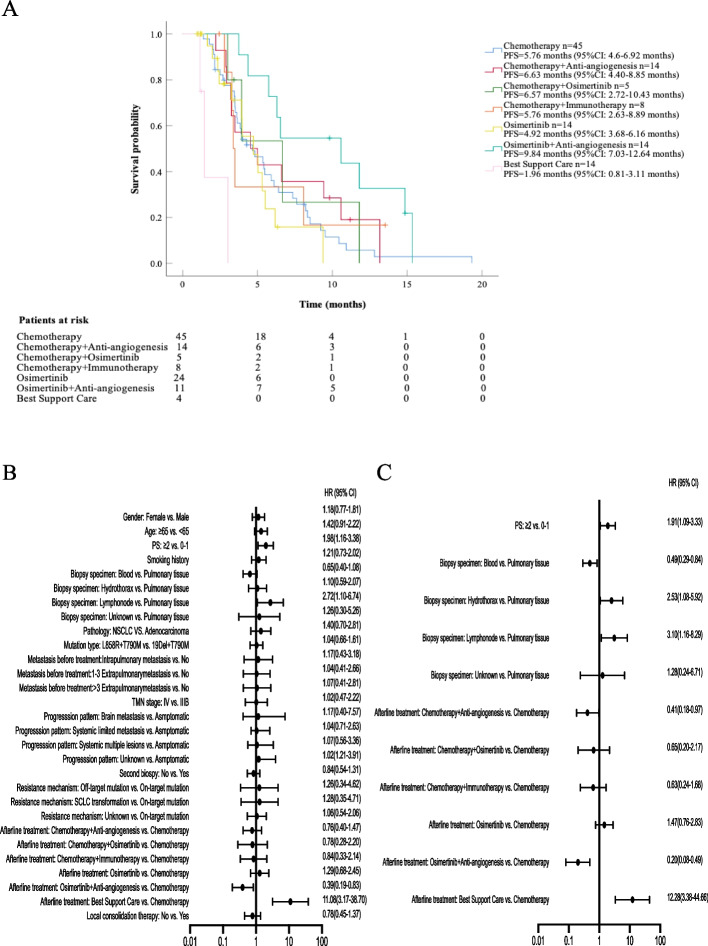
The analysis of mPFS in different post-osimertinib treatment groups. **A** The mPFS stratified by different post-osimertinib treatment. **B** Univariate COX regression analysis by baseline characteristics for mPFS. **C** Multivariate COX regression analysis by baseline characteristics for mPFS

The median duration of follow-up time was 7.6 months. The mPFS duration of and the Kaplan-Meier survival curves of patients undergoing various treatment patterns after osimertinib failure are displayed in Fig. 2A. The mPFS among 111 patients was 6.3 months (95% CI 5.4–7.2 months), and a statistically significant difference was confirmed among the groups (*P* < 0.001). Specifically, the longest mPFS was 9.84 months (95% CI 7.0–12.6 months) in the osimertinib plus anti-angiogenesis group, and significantly longer than that in the chemotherapy group (5.76 months, 95% CI 4.6–6.92 months, *P* = 0.012), the osimertinib monotherapy group (4.92 months, 95% CI 3.68–6.16 months, *P* = 0.003), and the BSC group (1.96 months, 95% CI 0.81–3.11 months, *P* < 0.001). Moreover, the mPFS of chemotherapy plus anti-angiogenesis was significantly superior to that of chemotherapy plus immunotherapy (6.63 vs*.* 5.76 months, *P* = 0.003). To further understand the predictive value of clinical parameters for treatment efficacy, we conducted Cox regression analyses of clinicopathological characteristics associated with mPFS after osimertinib failure using the Cox proportional hazards model. All variables with *P* < 0.1 in the univariate analysis were further included in the multivariate analysis. The related Cox regression data are presented in Additional file 1: Table S3 and Fig. 2B. Multivariate analysis demonstrated that ECOG PS ≥ 2 (vs*.* ECOG PS 0–1, *P* = 0.02), lymph node biopsy specimens (vs*.* pulmonary tissue, *P* = 0.02), and BSC as subsequent therapy after osimertinib resistance (vs*.* chemotherapy, *P* < 0.01) were independently associated with shorter mPFS. Conversely, chemotherapy plus anti-angiogenesis as subsequent therapy after osimertinib resistance (vs*.* chemotherapy, *P* < 0.01) was independently associated with a longer mPFS (Fig. 2C).

**Fig. 3:**
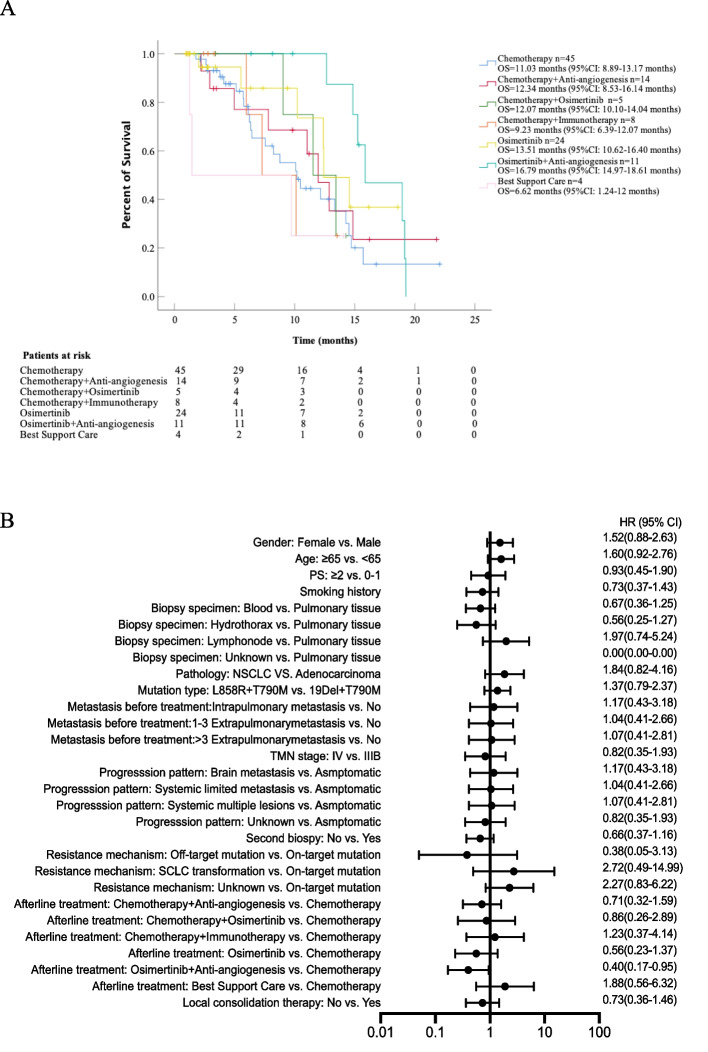
The analysis of mOS in different post-osimertinib treatment groups. **A** The mOS was stratified by different post-osimertinib treatments. **B** Univariate COX regression analysis by baseline characteristics for mOS

The mOS duration of each patient and the Kaplan-Meier survival curves of patients undergoing diverse treatment patterns after osimertinib failure are presented in Fig. 3A. The median mOS among 111 patients was 12.3 months (95% CI 10.9–13.6 months), and no statistically significant difference was found among the groups (*P* = 0.23). Similarly, the longest mOS was 16.79 months (95% CI 14.97–18.61 months) in the osimertinib combining anti-angiogenesis group, and significantly better than that in the chemotherapy group (11.0 months, 95% CI 8.9–13.2 months, *P* = 0.026), the chemotherapy plus osimertinib group (12.1 months, 95% CI 10.1–14.0 months, *P* = 0.021), the chemotherapy plus immunotherapy group (9.2 months, 95% CI 6.4–12.1 months, *P* = 0.006), and the BSC group (6.6 months, 95% CI 1.24–12.0 months, *P* = 0.004). Similarly, Cox regression analysis was performed for mOS (Additional file 1: Table S4 and Fig. 3B). However, none of the parameters showed a predictive effect on mOS in our cohort.

### Osimertinib combined with anlotinib exhibited promising efficacy in xenograft mice models

**Fig. 4:**
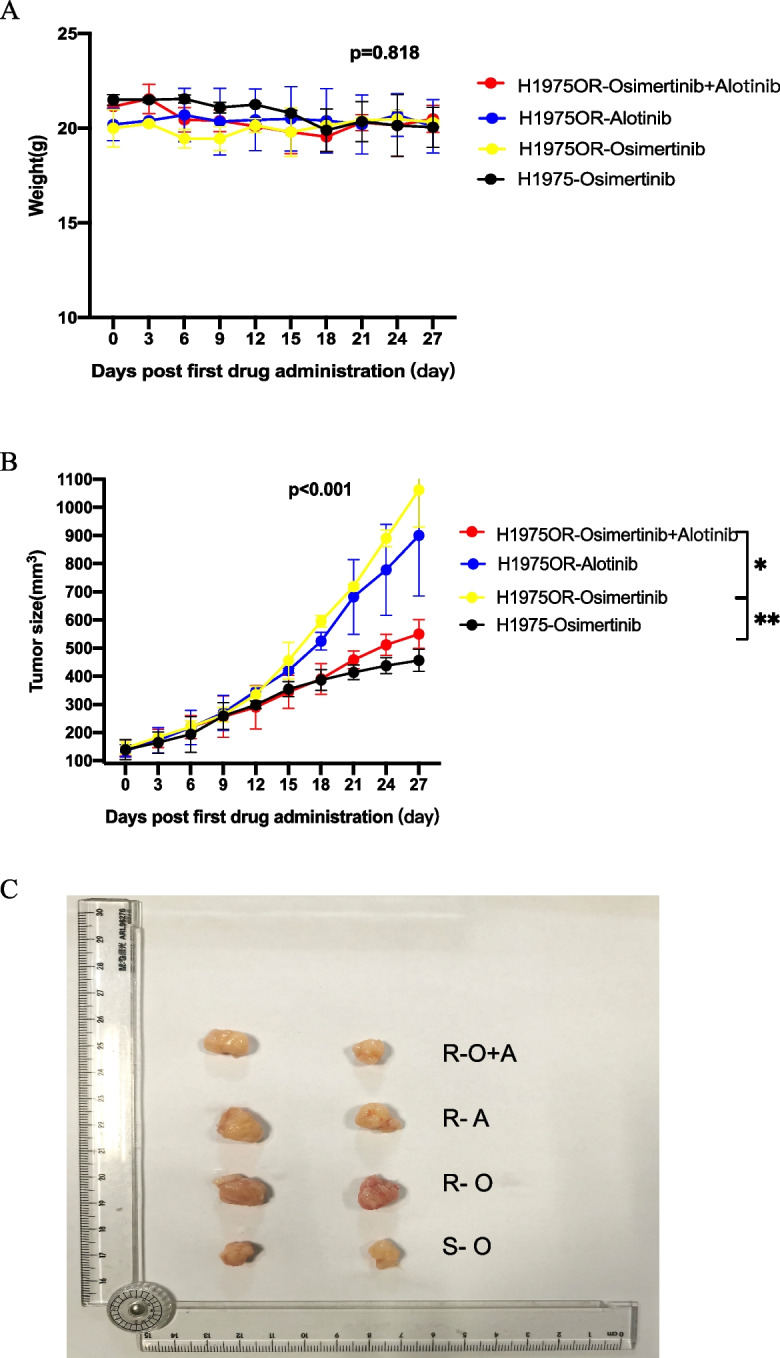
Efficacy of osimertinib combined with anlotinib for osimertinib-resistant xenografts in vitro. **A** The weight curve of humanized mice in each group during treatment. **B** The tumor volume curve of humanized mice during treatment. **C** Measurement of fresh xenograft tumor tissues after the mice were euthanized at the end of treatment (*, *P*<0.05; **, *P*<0.01)

Humanized mice were established and checked for the percentage of hCD45^+^ in peripheral blood before tumor xenograft (Additional file 2: Fig S1). Tumor-bearing mice generally grew well without GVHDs and significant weight loss of body weight among the groups (Fig. 4A). Compared to the osimertinib-sensitive H1975 engraft (*n*=2), the growth inhibitory effect of osimertinib on drug-resistant H1975OR tumors (*n*=2) was significantly weakened (*P*<0.01) (Fig. 4B,C). In addition, the efficacy of osimertinib combined with anlotinib in H1975OR tumors (*n*=2) was significantly more robust than that of osimertinib alone (all *P*<0.05), indicating that combination therapy could effectively inhibit the growth of osimertinib-resistant tumors (Fig. 4B,C).

### Alterations in host immunity with the combinational treatment of osimertinib and anlotinib

**Fig. 5:**
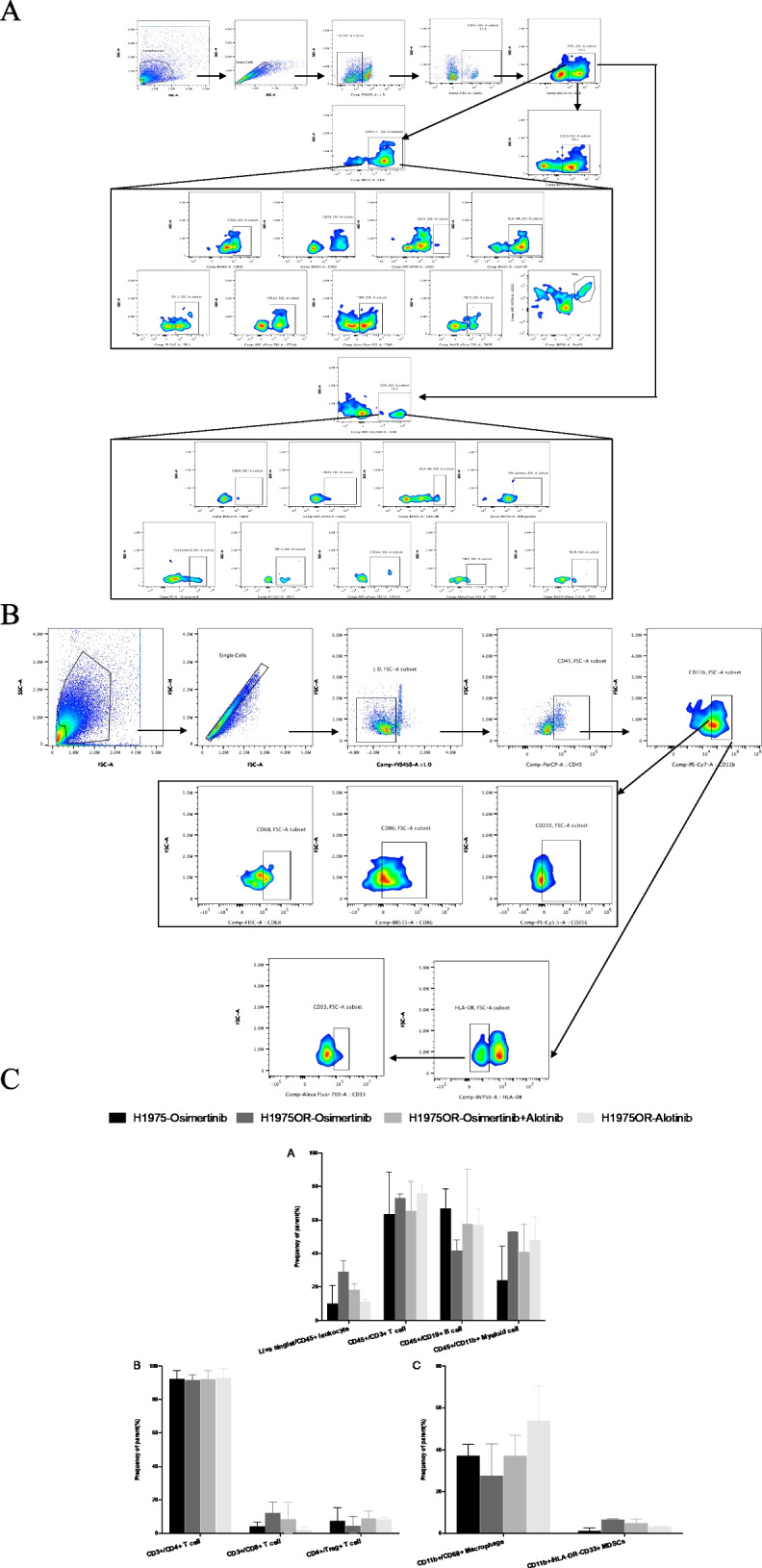
Flow cytometry of immune cells in peripheral blood of mice demonstrated a successful immune reconstitution. **A** Gating strategy of lymphocytes including T cells and B cells. Total leukocytes were gated with CD45^+^, in total lymphocytes were gated with CD3^+^. Then the CD4^+^ helper T cells, CD8^+^ cytotoxic T cells, and CD19^+^ B cells were then delineated in CD3^+^ lymphocytes, respectively. Next, CD4^+^ T cells were further divided into early activation (CD69^+^), middle activation (CD25^+^), late activation (HLA-DR^+^); exhausted (PD1^+^/CTLA-4^+^/TIM3^+/^TIGIT^+^), as well as immunosuppressive Treg (FOXP3^+^CD25^+^) T cells. Similarly, we also applied the above gating strategy in CD8^+^T cells. In addition, we checked IFN-γ and Granzyme B, which were related to killing functions. **B** Gating strategy of myeloid cells involving macrophages and MDSCs. Among CD45^+^ leukocytes, overall myeloid lineage cells were delineated with CD11b^+^. Total macrophages were gated with CD68^+^; classically activated M1 macrophages were gated with CD86^+^; conditionally activated M2 macrophages were gated with CD206^+^; and immunosuppressive MDSCs were circled with HLA-DR-CD33^+^. **C** Comparison of the proportion of major cell components of tumor-infiltrating immune cells in each treatment group

To clarify the differences in overall immune construction between the osimertinib treating osimertinib-resistant engraft tumors (R-O) group (*n*=2) vs. the osimertinib treating osimertinib-sensitive engraft tumors (S-O) group (*n*=2), and the osimertinib plus anlotinib treating the osimertinib-resistant engraft tumors (R-O+A) (*n*=2) group vs. the R-O group, we analyzed the phenotype and function of T cells, macrophages, and MDSCs in the peripheral blood (Fig. 5A). Overall, there were no significant differences in primary immune cells, including CD45^+^ cells, CD3^+^ T cells (including CD4^+^ T cells, CD8^+^ T cells, and Foxp3^+^CD25^+^ Tregs), CD19^+^ B cells, and CD11b^+^ myeloid cells (including CD68^+^ macrophages and HLA-DR^−^CD33^+^ MDSCs) among the groups (Fig. 5B). The proportion of middle-activated CD25^+^CD8^+^ T cells was significantly higher in the S-O group than in the R-O group (*P<*0.05) and R-A group (*P<*0.05), but was statistically equivalent between the S-O group and the R-O +A groups (Additional file 2: Fig S2A). Meanwhile, there was no difference in the levels of secreted IFN-γ, granzyme B, and the expression levels of immune checkpoints on CD8^+^ T cells among the groups (Additional file 2: Fig S2A). No significant differences were observed between the M1 and M2 type macrophages (Additional file 2: Fig S2B).

**Fig. 6:**
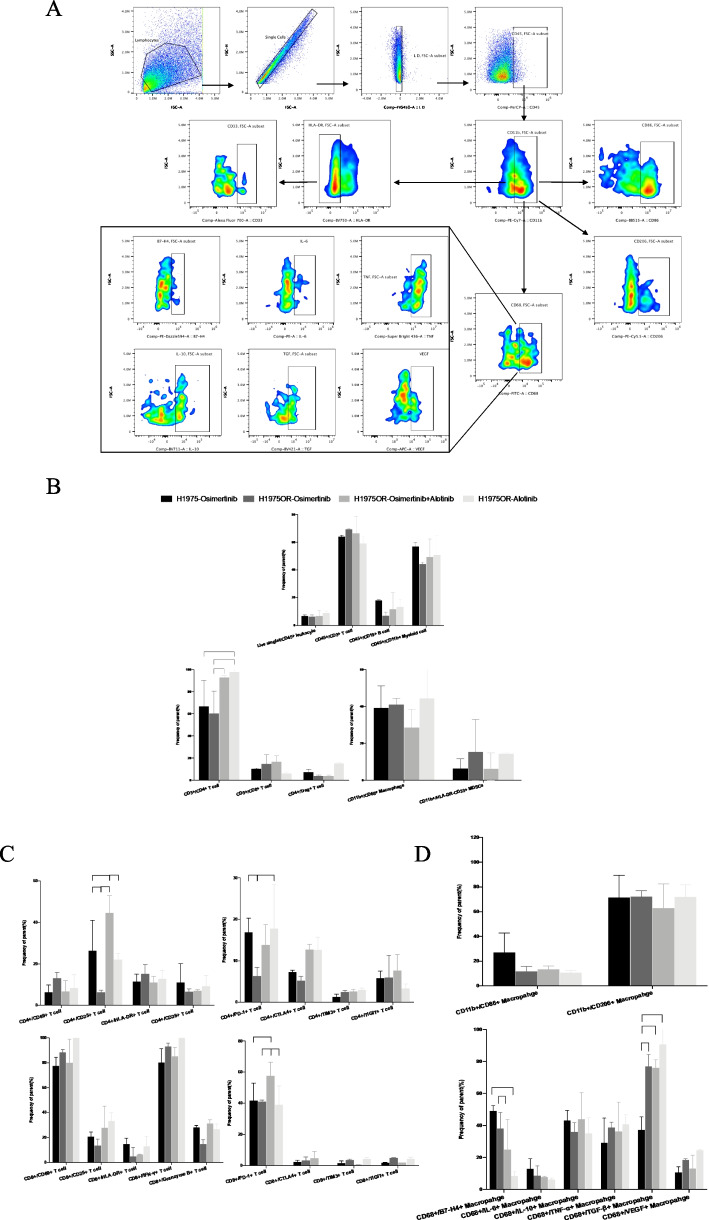
Flow cytometry detection of infiltrating immune cells in tumors. **A** In addition to the panel used in blood, we further detected cytokines secreted by CD68+ macrophages in tumors. **B** Analysis of tumor-infiltrating immune cells. **C** Comparative analysis of tumor-infiltrating T cells. **D** Tumor-infiltrating macrophages and their functional analysis

Furthermore, we detected the infiltration of immune cells into the tumor microenvironment. The T cell panel design and gating strategy were the same as those in the peripheral blood (Additional file 2: Fig S3). Here, we additionally detected the cytokines secreted by CD68^+^ macrophages, including IL-6, TNF-α, IL-10, TGF-β, and VEGF (Fig. 6A). The results are shown in Fig. 6B–D. Compared to the S-O group, the proportion of CD25^+^ middle-activated, PD1^+^ exhausted CD4^+^ T cells was significantly decreased (all *P<*0.05), and TGF-β secretion by CD68^+^ macrophages were significantly increased (*P<*0.001) in R-O group. When compared with the R-O group, combination therapy significantly increased the infiltration of CD4^+^ T cells (*P<*0.05), CD25^+^ activated CD4^+^ T cells (*P*<0.001), and the expression of PD-1 on CD8^+^ Tc cells (*P<*0.05) in the R-O+A group. There were no differences in the other cell proportions or markers of activation, exhaustion, and function of T cells and macrophages among the groups.

### Cellular components and functional annotation in humanized mice xenograft tumors

**Fig. 7:**
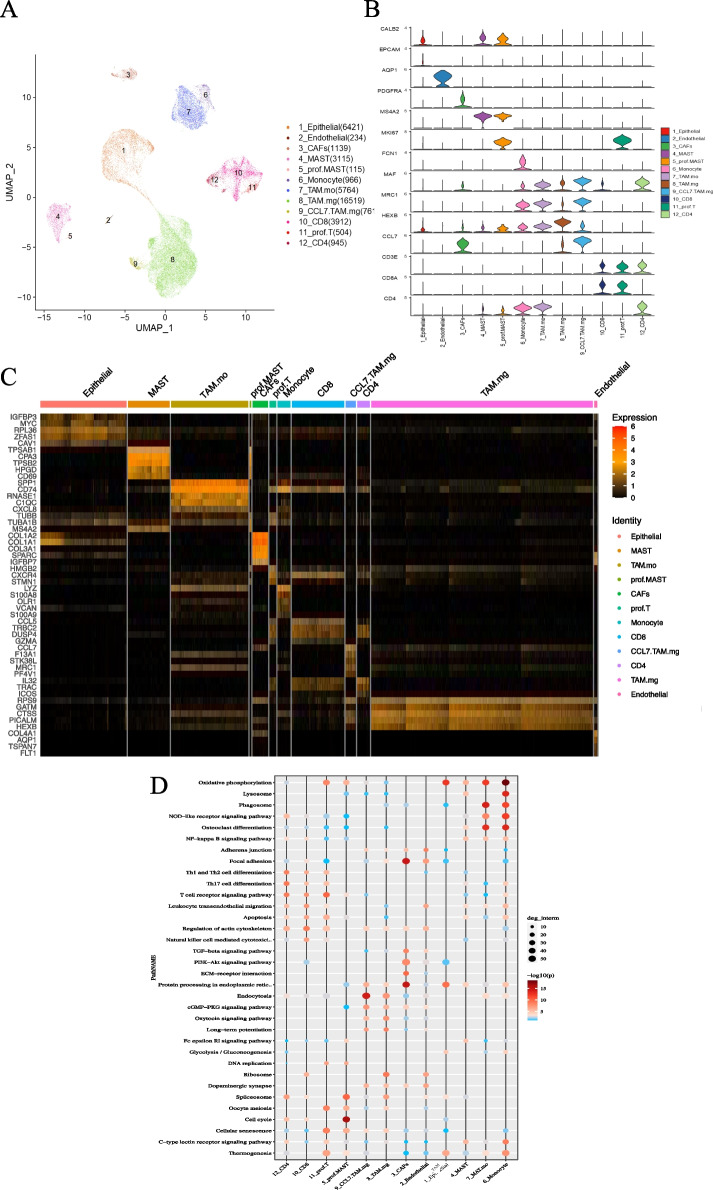
Cell type annotation and function enrichment analysis by scRNA sequencing of tumor tissues. **A** Cell Clusters Visualization by UMAP dimension reduction analysis. **B** Specific markers for cell annotation. **C** Heatmap of Top5 differential genes between cell types. **D** Function enrichment analyses on DEGs

UMAP plots demonstrated that 40,395 cells from humanized mouse xenograft tumors could be clustered into 12 cell subsets by dimensionality reduction (Fig. 7A). These are (epithelial), endothelial (endothelial), cancer-associated fibroblasts (CAFs), mast cells (MAST), proliferating mast cells (prof. MAST), and monocyte-like macrophages (TAM. mo), microglia-like macrophages (TAM.mg), microglia-like macrophages with high CCL7 expression (CCL7.TAM.mg), CD8^+^ T cells, and proliferating T cells (pro. T) and CD4^+^ T cells. TAM account for the most significant proportion of tumor-infiltrating immune cells. Specific marker genes for the cell clusters are shown in Fig. 7B.

Top5 differential expression genes (DEGs) in clusters were screened and are shown in Fig. 7C. GO and KEGG pathway enrichment analyses were performed on these DEGs, and most cell functions were similar to those in previous reports (Fig. 7D). Here, we highlighted the functional enrichment of TAM.mg and CCL7. Ribosomes, the cGMP-PKG pathway, the oxytocin signaling pathway, long-term potentiation, and endocytosis were significantly upregulated in TAM.mg. However, MAST, TAM.mo, and monocytes are mainly enriched in phagosome formation, osteoclast differentiation, oxidative phosphorylation, and NOD-like receptor signaling pathways, which are closely related to the recognition of pathogen-associated molecular patterns (PAMPs). Thus, TAM.mg and CCL17.TAM.mg and CCL7.TAM.mg were distinguishable from classical monocytes and macrophages, which seemed to play a similar promoting function on neural pathways with microglia.

### Cellular compartment, function, and communication dynamics between the R-O vs. the S-O groups and the R-O+A vs. R-O group

**Fig. 8:**
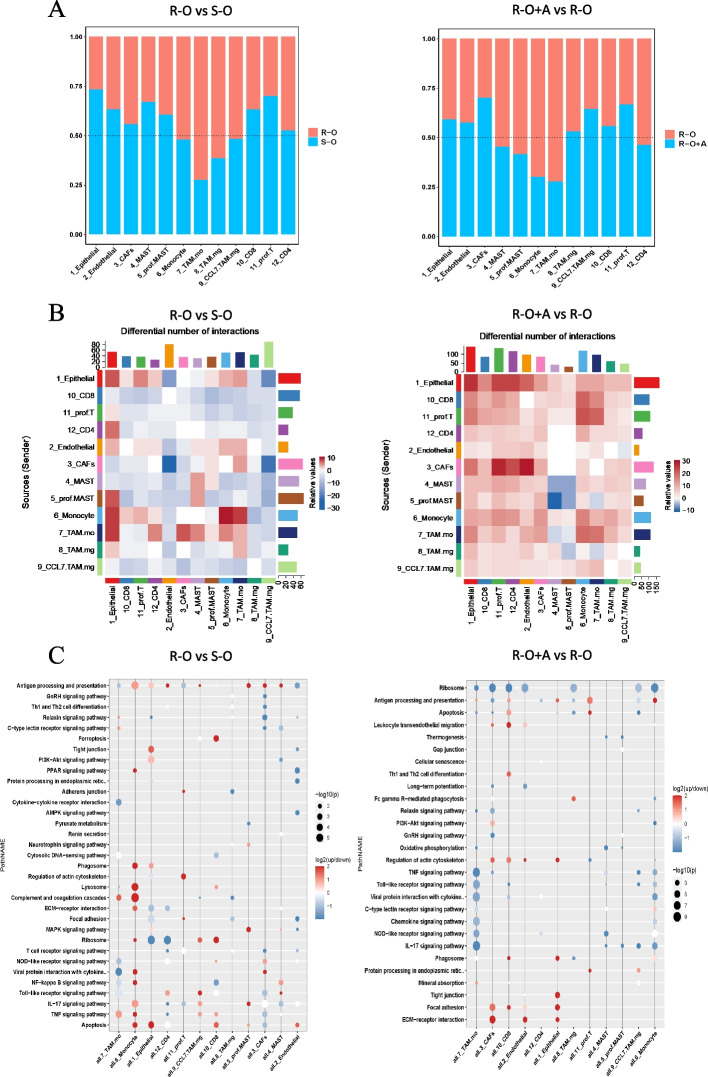
Differences in TME between R-O and S-O groups and between R-O+A and R-O groups. **A** The relative abundance of cell components was compared. **B** Differences in cell communication among the above cells. **C** Differences in function enrichment in those cell types

Regarding the cell components between the R-O and S-O groups, CD4^+^ T, CD8^+^ T, and pro. T in the S-O group were more abundant than those in the R-O group, whereas all types of TAMs in the R-O group were higher than those in the S-O group (Fig. 8A). With the addition of anlotinib, CD8^+^ T cells, pro. T, CCL7_TAM.mg in the R-O+A group was higher than that in the R-O group, while TMA.mo and monocyte were lower (Fig. 8A).

Regarding intercellular communication, TAM.mo and monocytes showed increased communication with other cells. Interactions of T cells with other cells were decreased in the R-O group compared to those in the S-O group (Fig. 8B). Comparing cell communications of the R-O+A and R-O groups, most cell-to-cell interactions were upregulated in the R-O+A group (Fig. 8B).

Compared to the S-O group, the DEGs of monocytes in the R-O group were mainly upregulated and involved in the complement and coagulation cascades, lysosomal components, antigen processing, presentation functions, and IL-17 signaling pathways (Fig. 8C). Downregulated DEGs in the R-O group were mainly enriched in the ribosome, focal adhesion, ECM receptor interaction (epithelial), lysosome (CD4^+^ T), and viral proteins interacting with cytokines (TAM.mo) (Fig. 8C). However, the number of DEGs, mainly in TAM.mo, was downregulated in the R-O+A group compared with that in the R-O group (Fig. 8C). The DEGs of macrophages were mainly enriched in the TNF and IL-17 signaling pathways, Toll-like receptors, NOD-like receptors, and C-type lectin receptor signaling pathways associated with innate immune PAMP recognition. A similar downward trend was observed for monocytes. On the other hand, the upregulated DEGs in the R-O group were mainly enriched in the ECM receptor function and tight junction function of CAFs and epithelial cells and the antigen processing and presentation of the pro. T cells (Fig. 8C).

As mentioned above, the differential proportion and function of immune cells in the TME between the different groups were mainly concentrated in T cells and TAMs. Thus, we further analyzed the subtypes of T cells and TAMs.

### The combination of osimertinib and anlotinib promoted cytotoxic T cell infiltration

**Fig. 9:**
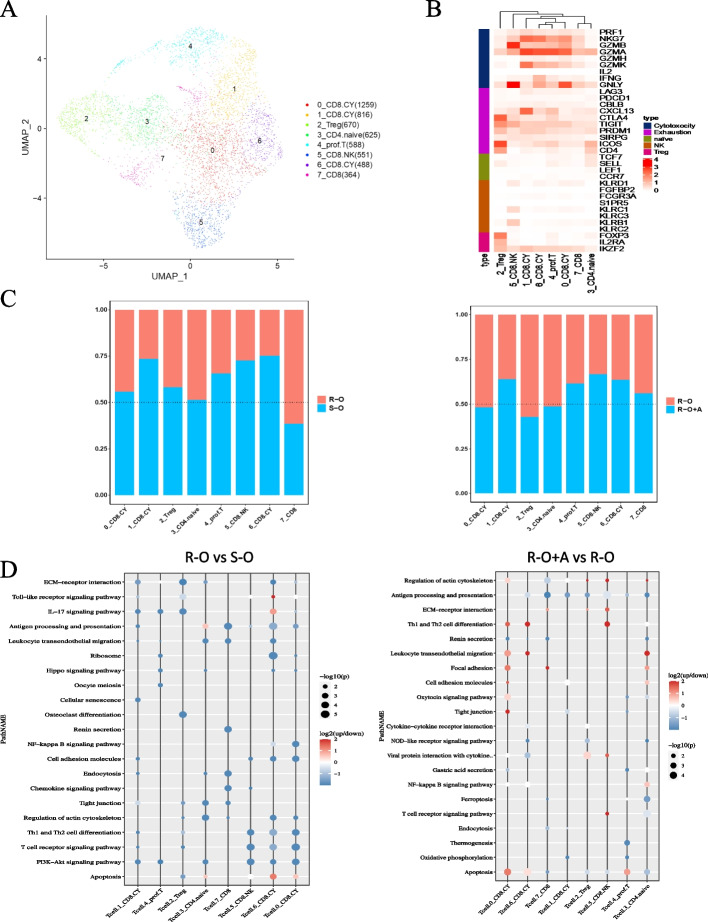
T cells subtype analysis between R-O and S-O groups and between R-O+A and R-O groups. **A** T Cell subtype cluster visualization by UMAP dimension reduction analysis. **B** Function clusters of specific markers for subtype annotation. **C** The relative abundance of various subtype T cell components was compared. **D** Differences in function enrichment were noted in those cell subtypes

T cells were further clustered into eight subtypes according to their specific marker genes (Fig. 9A) and could be classified into five categories: cytotoxic, exhausted, naive, NK, and Treg (Fig. 9B). Interestingly, all annotated subtypes of T cells in the R-O group were fewer than those in the S-O group (Fig. 9C), whereas with the combination of anlotinib, the number of 1_CD8. CY, 6_CD8. CY, CD8. The number of NK and CD8 cells in the R-O+A group was higher than that in the R-O group (Fig. 9C). Compared to the S-O group, the functional enrichment of DEGs in the R-O group was mainly downregulated in CD8. NK, 0_CD8. CY and 6_CD8. CY were mainly enriched in the T cell receptor signaling pathway, Th1 and Th2 cell differentiation, and PI3K-Akt signaling pathways (Fig. 9D). Meanwhile, the upregulated DEGs in the R-O+A group were mainly related to apoptosis, leukocyte transendothelial migration, tight junctions, and other functions of 0_CD8. The CY subtype was compared with the R-O group (Fig. 9D).

### Suppressive immune microenvironment primed by macrophages

**Fig. 10:**
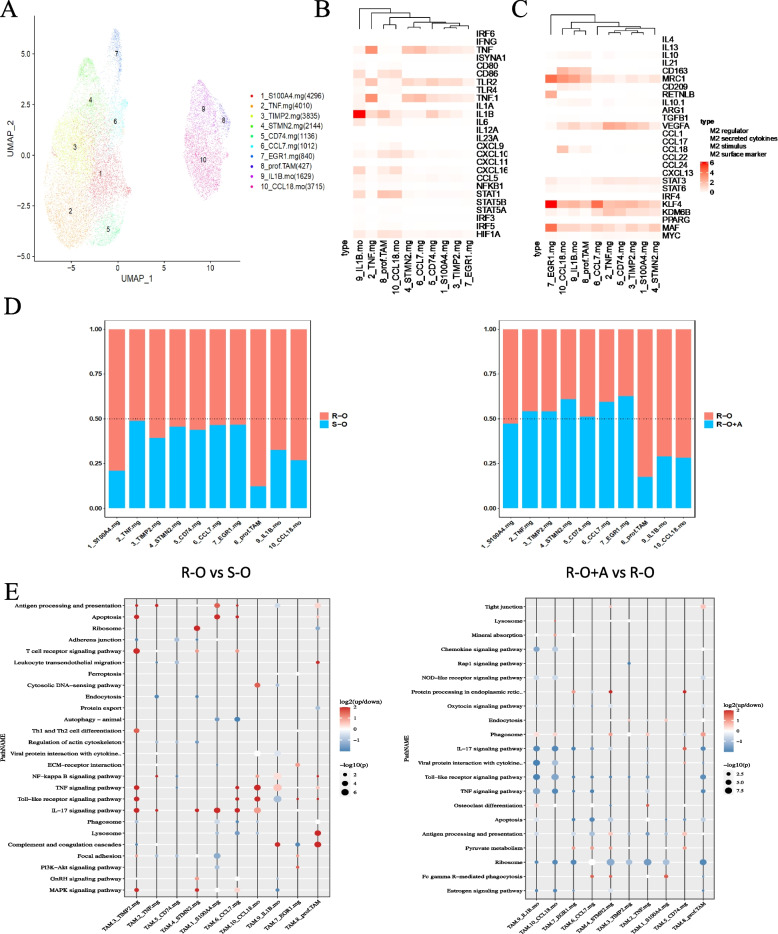
Macrophage subtype analysis between R-O and S-O groups and between R-O+A and R-O groups. **A** Macrophage subtype cluster visualization by UMAP dimension reduction analysis. **B** Function clusters of specific markers for M1 type macrophage annotation. **C** Function clusters of specific markers for M2 type macrophage annotation. **D** The relative abundance of various subtype macrophages was compared. **E** Differences in function enrichment in those cell subtypes were also noted

Macrophages clustered into ten subtypes (Fig. 10A), according to four specific marker genes: regulation, cytokine secretion, cell stimulation, and surface markers (Fig. 10B,C). The proportions of monocyte-derived IL1β secreting TAM (IL1β.mo) and monocyte-derived CCL18 secreting TAM (CCL18.mo) in the R-O group were higher than those in the other three groups. These two subtypes have low expression of the M1 surface marker (CD86) and high expression of M2 surface markers (MRC1 and CD163) (Fig. 10B,C). Moreover, we noted that they were both highly expressed along with VEGFA.

Furthermore, we compared the proportion and function of macrophage subtypes between the two groups (R-O vs. S-O, R-O+A vs. R-O). The number of macrophage subsets in the R-O group was higher than that in the S-O group, especially proliferating (pro.TAM) microglia-like macrophages with a high expression of S100A4 (S100A4.mg), CCL18.mo, and IL-1β.mo (Fig. 10D). We also noted that the number of pro.TAM, CCL18.mo, and IL-1β.mo in the R-O+A group were lower than those in the R-O group (Fig 10D).

Furthermore, compared to the S-O group, the upregulated DEGs on pro.TAM in the R-O group were related to the lysosome, complement, and coagulation cascade. The upregulated DEGs of S100A4.mg and CCL18.mo were mainly related to the IL-17 signaling pathway, whereas upregulated DEGs were related to CCL18.mo and IL-1β.mo was associated with TNF signaling (Fig. 10E). When comparing the R-O+A group with the R-O group, the DEGs were mainly downregulated and enriched in IL-17, toll-like receptors, and TNF signaling pathways. In addition, the downregulated DEGs in other macrophage subsets were related to lysosomes (Fig. 10E).

## Discussion

Despite the promising clinical efficacy of osimertinib in treating patients with T790M mutations, patients may also develop resistance due to various mechanisms [30]. So far, strategies to overcome osimertinib resistance include using anti-EGFR monoclonal antibodies, administering fourth-generation EGFR-TKIs, or combining osimertinib with other specific inhibitors based on individual resistance mechanisms [31]. However, this NGS-based management of osimertinib resistance may not always be accessible in real-world treatment, and re-biopsy may not always find therapeutic targets. Studies on post-osimertinib treatment and survival are urgently needed. Cheng et al. performed a retrospective analysis of 89 patients who received second- and further-line osimertinib, revealing that targeted therapy after osimertinib progression did not significantly prolong the pPFS and pOS compared to chemotherapy [32]. TKI plus anti-angiogenesis therapy displayed longer PFS than chemotherapy, osimertinib monotherapy, or BSC in the present study. Combination therapy also showed longer OS than chemotherapy, chemotherapy plus osimertinib, chemotherapy plus immunotherapy, and BSC. Moreover, this study also found that patients who underwent re-biopsy had significant PFS (3.5 vs. 3.3 months, *P*=0.016) and OS (11.7 vs. 6.8 months, *P*=0.013), especially those with targetable markers and matched treatment. In our study, 41 (36.9%) patients underwent re-biopsy after osimertinib resistance. No significant PFS or OS advantage was displayed compared to patients who did not undergo re-biopsy. Cox regression analysis also revealed that re-biopsy was not a predictor of PFS (*P*=0.45) or OS (*P*=0.4). Obtaining the biopsy from metastatic lymph nodes was found to be significantly linked to a shorter PFS whereas obtaining from blood was found to be associated with statistically significant improvements, which could be explained based on the characteristics and nature of the two biopsy methods. We demonstrated that osimertinib plus anti-angiogenesis therapy could confer survival benefits after osimertinib resistance.

EGFR and VEGFR share common downstream signaling pathways, and EGFR activation has been shown to improve VEGF expression in tumors [33, 34]. This forms the basis of combination therapy targeting the EGFR/VEGFR axis. Attempts have been made to explore whether the combination of EGFR-TKIs and anti-angiogenesis therapy could improve the prognosis of EGFR-mutant mutations. A multicenter, double-blind, phase III RELAY trial (NCT02411448) demonstrated a synergistic effect of this EGFR and VEGFR inhibitor combination (19.4 months vs. 12.4 months) [35]. However, several phase II trials showed no significant difference in PFS between osimertinib plus bevacizumab and osimertinib monotherapy either in first-line or second-line treatment of EGFR-mutant NSCLC patients [36–38]. The potential clinical efficacy of anti-angiogenic therapy in osimertinib-acquired resistance patients remains unclear. Anlotinib is a multi-target TKI that targets VEGFR, FGFR, PDGFR, and c-kit [39]. The phase II ALTER-L031 (NCT04136535) trial is ongoing to evaluate the efficacy of anlotinib combined with chemotherapy in EGFR-mutant patients with disease progression to osimertinib [10]. Furthermore, a combination of anlotinib, pemetrexed, and toripalimab was assessed in T790M-positive patients after osimertinib resistance in another trial (NCT04316351) [10]. Based on the results of a phase III ALTER 0303 trail, anlotinib has been approved for the treatment of advanced NSCLC in the third line or beyond in China. Recently, a multicenter retrospective study indicated a survival benefit of anlotinib in NSCLC patients that acquired osimertinib resistance [40]. Furthermore, this study further conducted in vitro study that anlotinib in combination with osimertinib showed significantly strong inhibitory effect on osimertinib-resistant NCSCLC cell lines than anlotinib or osimertinib alone. Consistent with the above study, we also observed sound therapeutic effects in osimertinib-resistant NSCLC patients with anlotinib plus osimertinib treatment pattern. These findings suggest that anlotinib combined with osimertinib may be effective for osimertinib-resistant NSCLC. Furthermore, we attempted to determine the rationale for these combinations from the perspective of changes brought to the tumor microenvironment.

Hypoxia is an essential feature of the tumor microenvironment. Tumors need to establish a new blood supply to meet their need for oxygen and nutrients, which leads to increased VEGF secretion by tumors and promotes endothelial cell proliferation and angiogenesis by engaging VEGFR2 [41]. However, tumor-associated blood vessels typically acquire aberrant morphology, mainly characterized by disorganized branches, discontinuous endothelial cell lining, insufficient basement membrane coverage, and abnormal lumens, which are conducive to abnormal function [42]. Tumor cells can adapt to these conditions via several mechanisms [43]. In contrast, abnormal vessels restrict the entry of drugs and immune cells into tumors and impair their effectiveness after infiltration [44]. Neovascular networks have been reported to increase tumor heterogeneity, facilitate tumorigenesis, and promote drug-resistant phenotypes. We also found that adding an anti-angiogenesis drug to osimertinib could improve the prognosis of patients with osimertinib resistance. From this perspective, we hypothesized that normalizing the tumor vasculature in the TME may help overcome the resistance to osimertinib. Several preclinical studies have highlighted the role of anti-angiogenesis therapy in the antitumor immune response [45]. Several possible mechanisms have been elucidated for the immunomodulatory effect of VEGF/VEGFR blockage, such as increasing T cell infiltration, promoting DC maturation, enhancing T cell priming, and decreasing Treg infiltration [46]. Combining VEGF/VEGFR inhibitors with osimertinib may represent a new option for patients with EGFR mutations in whom TKIs have failed. In the present study, we performed FACS and scRNA-seq to investigate the effect of osimertinib on the tumor microenvironment.

T cells are essential cells that accomplish an antitumor immune response. The role of VEGF in modulating T cell infiltration and function has been extensively investigated in preclinical models [47]. Excessive levels of VEGF can inhibit T cell trafficking, proliferation, and function in the tumor microenvironment. Moreover, VEGF can hamper T cell activation by inhibiting DC maturation and antigen presentation [48, 49]. In our findings, we observed that the R-O group exhibited a significant decrease in functional T subtype cells, primarily consisting of cytotoxic T cells, compared to the S-O group. The R-O group also showed reduced TCR signaling and Th cell differentiation-related signaling. Combination treatment with anlotinib increases the proportion of CD8^+^ cells. T_EM_、CD8. T_EFF_、CD8. NK cells and a decreased proportion of Tregs in osimertinib-resistant tumors. The above results indicate that the O+A treatment pattern could restore the infiltration level of cytotoxic T cells and enhance the antitumor immune response by vascular normalization.

Tissue macrophages have two main origins. One group of macrophages originates from tissue-resident myeloid cells, which differentiate into progenitor cells that migrate early into the tissue. The other group originates from bone marrow stem cells, which differentiate into marrow-derived monocytes and extravasate into tissues via circulation [50]. In our humanized mouse cancer model, macrophages were derived from monocytes in circulation that migrated to the tumor via gradients of soluble chemoattractants. After infiltration, monocytes differentiate into TAMs under tumor-derived cytokines and chemokines that sculpt their phenotype, which helps TAMs gain molecular and functional heterogeneity [50]. TAMs can be polarized into pro-immature M1 and pro-tumoral M2 phenotypes. However, the phenotype and function of TAM are plastic and dynamic in a continuum of states [51].

UMAP analysis in our scRNA-seq divided the TAMs into two major clusters. Based on their specific expression markers and functions, we annotated them as monocyte-like TAM.mo, microglia-like TAM.mg, and the latter type, commonly found in gliomas. Muller et al. found that brain-resident TAMs have a different gene expression profile than blood-derived TAMs in an scRNA-seq assay of human glioma tissues. Monocyte-derived TAMs mainly accumulate in the perivascular area with M2 function and metabolic phenotype and are negatively correlated with patient prognosis [52]. Chavez et al. found that primary TAMs in tissues can recruit monocytes by secreting chemokines such as CCL7, and these monocytes can promote TAMs polarization via different cytokines [53]. Our functional enrichment analysis of TAM.mo and TAM.mg cluster cells was similar to that reported previously. Thus, TAM.mo may be closely related to the acquisition of the M2 TAM phenotype. These findings may explain the sequencing results.

Regarding the proportion of cell-type compositions, TAM.mo was higher, but TAM.mg was lower in the R-O group than in the S-O group. These changes were reversed in the combined treatment of R–O+A group. We speculated that monocytes in the circulatory system were recruited to tumors and then underwent subtype conversion, mainly from the TAM.mo subtype to TAM.mg. Compared with the S-O group, the TAM.mo to TAM.mg subtype conversion process was reduced in the R-O group, leading to the accumulation of tumor-promoting TAM.mo subtypes. However, the combination of osimertinib and anlotinib reinitiated the process of subtype conversion and inhibited the growth of drug-resistant tumors. Further subtype analysis divided TAM.mo into two clusters, which we annotated as CCL18.mo and IL1β.mo. The CCL18.mo subtype, which highly expresses MRC1 receptors and secretes CCL18 molecules, is a typical M2-type immunosuppressive cell type. It is closely related to immunosuppressive spatial reprogramming in the metastatic cancer microenvironment [54]. The IL1β.mo subtype secreting high levels of pro-inflammatory IL-1β molecules is of interest, demonstrating a superficially high expression of the M2-type markers (MRC1 and CD163) and the M2-type polarization regulatory genes (KLF4 and MAF). Therefore, we classified IL-1β.mo in the M2-type TAM phenotype [55]. Our results indicate that the increase in the above immunosuppressive TAM.mo subtypes was an important cause of osimertinib resistance. Combination with anlotinib reduced the proportion and downregulated functional genes of these TAM.mo subtypes, which might be the mechanisms underlying osimertinib resistance.

High levels of tumor-secreting VEGF also help to construct an immunosuppressive environment by promoting the recruitment and proliferation of immunosuppressive cells, including Treg cells, MDSCs, and M2-type macrophages [56]. Macrophages are the most common component of tumor mesenchymal cells, and many macrophages are associated with increased vasculature in tumors [57]. In addition, macrophages are an essential source of VEGFA within the tumor microenvironment, exerting a pro-angiogenic effect and facilitating tumor metastasis [58]. TAMs also produce pro-inflammatory cytokines such as TNF, IL-1β, IL-6, and FGF2 [59]. In addition, the intimate physical association between TAMs and endothelial cells may facilitate the infiltrative growth of tumor-associated blood vessels via ECM degradation, endothelial cell activation, and migration [60]. Functional analysis of intratumoral TAMs showed that TAM could secrete VEGFA and IL-1β molecules to promote angiogenesis. Blocking the VEGFA-VEGFR angiogenic pathway by combining anti-angiogenic drugs may be essential for reversing drug resistance.

Our study has some limitations. First, this was a single-center retrospective study with relatively limited samples, selection bias may be inevitable, and further large-scale, multicenter prospective studies are needed. Second, we lacked a detailed report of NGS tests for patients who underwent re-biopsy, which may hinder our precise interpretation of post-osimertinib treatment outcomes, more comprehensive analysis of genomic profiles was needed. Additionally, the sample size in our in vivo experiment was small owing to the cost and difficulty of constructing humanized mice, which may cause bias in the results. Finally, although we suggested potential mechanisms of the combination pattern to overcome osimertinib resistance, experimental validation is lacking. More efforts should be made to clarify these specific mechanisms in the future.

## Conclusions

Osimertinib plus anlotinib could improve the prognosis of patients with a progressed disease on second-line osimertinib treatment, which may ascribe to increased T cell infiltration and TAM remodeling via VEGF-VEGFR blockage.

## Supplementary Information

**Additional file 1:** **Table S1.** COX regression analysis by baseline. **Table S2.** Univariate Cox regression analysis by baseline characteristics for OS. **Table S3.** Antibodies for panel A for T cells. **Table S4.** Antibodies for panel A for macrophages and MDSC.

**Additional file 2:** **Fig S1.** Evaluation of humanized mouse model. A. Gating strategy for detecting hCD45^+^ leukocytes in peripheral cells in mice; B. Flow cytometry results of each humanized mice constructed in our experiment; C. Proportion of hCD45^+^/mCD45^+^ in peripheral blood. **Fig S2.** Flow cytometry detection of infiltrating immune cells in peripheral blood. Comparative analysis of tumor-infiltrating T cells (A) and macrophages (B). **Fig S3.** Gating strategy of lymphocytes in tumor-infiltrating lymphocytes.Total leukocytes were gated with CD45^+^, in total lymphocytes were gated with CD3^+^. Then the CD4^+^ helper T cells, CD8^+^ cytotoxic T cells, and CD19^+^ B cells were then delineated in CD3^+^ lymphocytes, respectively. Next, CD4^+^ T cells were further divided into early activation (CD69^+^), middle activation (CD25^+^), late activation (HLA-DR^+^); exhausted (PD1^+^/CTLA-4^+^/TIM3^+^/TIGIT^+^), as well as immunosuppressive Treg (FOXP3^+^CD25^+^) T cells. Similarly, we also applied the above gating strategy in CD8+T cells. In addition, we checked IFN-γ and Granzyme B, which were related to killing functions.

## Data Availability

The datasets used and analyzed during the current study are available from the corresponding author on reasonable request.

## Abbreviations

CDX: Cell-derived xenograft, CDX
EGFR: Epidermal growth factor receptor
GO: Gene ontology
KEGG: Kyoto Encyclopedia of Genes and Genomes
NSCLC: Non-small cell lung cancer
ORR: objective response rate
OS: Overall survival
PFS: Progression-free survival
scRNA-seq: Single-cell RNA sequencing
TAMs: Tumor-associated macrophages
TKIs: Tyrosine kinase inhibitors
TME: Tumor microenvironment
UMAP: Uniform manifold approximation and projection
VEGF: Vascular endothelial growth factor
