# Correction: Osimertinib in combination with anti-angiogenesis therapy presents a promising option for osimertinib-resistant non-small cell lung cancer

**DOI:** 10.1186/s12916-024-03644-0

**Published:** 2024-09-20

**Authors:** Ruoshuang Han, Haoyue Guo, Jinpeng Shi, Sha Zhao, Yijun Jia, Xiaozhen Liu, Yiwei Liu, Lei Cheng, Chao Zhao, Xuefei Li, Caicun Zhou

**Affiliations:** 1 Department of Medical Oncology, Shanghai Pulmonary Hospital, Tongji University School of Medicine, Shanghai, People’s Republic of China; 2 Department of Oncology, The First Affiliated Hospital of Army Medical University, Chongqing, People’s Republic of China; 3 Department of Lung Cancer and Immunology, Shanghai Pulmonary Hospital, Tongji University School of Medicine, Shanghai, People’s Republic of China

**Correction** **:** **BMC Med 22, 174 (2024)**

**https://doi.org/10.1186/s12916-024-03389-w**

The original article [1] mistakenly omitted Equal Contribution attribution for the first four authors – Ruoshang Han, Haoyue Guo, Jinpeng Shi, and Sha Zhao – due to an error from the production team that handled the manuscript.

The Equal Contribution attribution has since been restored.
