# Supplementary material for: Osimertinib in combination with anti-angiogenesis therapy presents a promising option for osimertinib-resistant non-small cell lung cancer
Source: BMC Med. 2024 Apr 24;22:174. doi: 10.1186/s12916-024-03389-w (PMC11040894; doi:10.1186/s12916-024-03389-w)
Supplement: Supplementary file 1 — Additional file 1: Table S1. COX regression analysis by baseline. Table S2. Univariate Cox regression analysis by baseline characteristics for OS. Table S3. Antibodies for panel A for T cells. Table S4. Antibodies for panel A for macrophages and MDSC. [file 12916_2024_3389_MOESM1_ESM.docx]

**Supplementary Table 1. Antibodies for panel A for T cells.**

| Location | Marker | Fluorescein | Brand | Category No. |
| --- | --- | --- | --- | --- |
| Surface | CD45 | FITC | Biolegend | 304026 |
|  | CD3 | BV570 | Biolegend | 300436 |
|  | CD19 | BV510 | BD Pharmingen | 562947 |
|  | CD4 | BB515 | BD Pharmingen | 564419 |
|  | CD8 | APC-Fire810 | Biolegend | 344764 |
|  | CD28 | BV480 | BD Pharmingen | 566110 |
|  | CD25 | APC-R700 | BD Pharmingen | 565106 |
|  | CD69 | BV650 | BD Pharmingen | 563835 |
|  | PD-1 | PE-Cy7 | BD Pharmingen | 561272 |
|  | CTLA-4 | APC-eFluor 780 | Thermo | 47-1529-42 |
|  | TIGIT | PerCP-eFluor 710 | Thermo | 46-9500-42 |
|  | TIM3 | Alexa Fluor532 | Thermo | 58-3109-42 |
|  | HLA-DR | BV421 | BD Pharmingen | 562804 |
| Intracellular | IFN-γ | BV750 | BD Pharmingen | 566357 |
|  | Granzyme B | PE | Thermo | 12-8896-42 |
|  | FoxP3 | BB700 | BD Pharmingen | 566526 |

**Supplementary Table 2. Antibodies for panel A for macrophages and MDSCs.**

| Location | Marker | Fluorescein | Brand | Category No. |
| --- | --- | --- | --- | --- |
| Surface | CD45 | PerCP | Biolegend | 304026 |
|  | CD3 | BV570 | Biolegend | 300436 |
|  | CD19 | BV510 | BD Pharmingen | 562947 |
|  | CD206 | PerCP-eFluor710 | Biolegend | 321122 |
|  | CD11b | PE-Cy7 | BD Pharmingen | 557743 |
|  | CD33 | AF700 | Biolegend | 303436 |
|  | B7-H4 | PE-Dazzle 594 | BD Pharmingen | 562785 |
|  | CD86 | BB515 | BD Pharmingen | 564544 |
|  | HLA-DR | BV750 | BD Pharmingen | 746912 |
|  | CD47 | BV480 | BD Pharmingen | 563740 |
| Intracellular | CD68 | FITC | Biolegend | 333806 |
|  | VEGF | APC | R&D Systems | IC2931A |
|  | IL-6 | PE | Biolegend | 501107 |
|  | TGF-β | BV421 | BD Pharmingen | 562962 |
|  | TNF-α | BV650 | BD Pharmingen | 502938 |
|  | IL-10 | BV711 | BD Pharmingen | 564050 |

**Supplementary Table 3. COX regression analysis by baseline characteristics for mPFS**

| Variables | | Univariate analysis | | | |  | Multivariate analysis | | | |
| --- | --- | --- | --- | --- | --- | --- | --- | --- | --- | --- |
|  |  | HR | 95%CI | | P |  | HR | 95%CI | | P |
| Gender | |  |  |  |  |  |  |  |  |  |
|  | Female vs. Male | 1.18 | 0.77 | 1.81 | 0.46 |  |  |  |  |  |
| Age | |  |  |  |  |  |  |  |  |  |
|  | ≥65 vs. <65 | 1.42 | 0.91 | 2.22 | 0.12 |  |  |  |  |  |
| PS | |  |  |  |  |  |  |  |  |  |
|  | ≥2 vs. 0-1 | 1.98 | 1.16 | 3.38 | 0.01 |  | 1.91 | 1.09 | 3.33 | 0.02 |
| Smoking history | |  |  |  |  |  |  |  |  |  |
|  | Yes vs. No | 1.21 | 0.73 | 2.02 | 0.47 |  |  |  |  |  |
| Pathology | |  |  |  |  |  |  |  |  |  |
|  | NSCLC-NOS vs. Adenocarcinoma | 1.40 | 0.70 | 2.81 | 0.34 |  |  |  |  |  |
| TNM stage | |  |  |  |  |  |  |  |  |  |
|  | IV vs. IIIB | 1.02 | 0.47 | 2.22 | 0.96 |  |  |  |  |  |
| Biopsy specimen | |  |  |  | 0.037 |  |  |  |  | 0.00 |
|  | Pulmonary tissue | 1.00 |  |  |  |  | 1.00 |  |  |  |
|  | Blood | 0.65 | 0.40 | 1.08 | 0.09 |  | 0.49 | 0.29 | 0.84 | 0.01 |
|  | Hydrothorax | 1.10 | 0.59 | 2.07 | 0.77 |  | 2.53 | 1.08 | 5.92 | 0.03 |
|  | Lymph node | 2.72 | 1.10 | 6.74 | 0.03 |  | 3.10 | 1.16 | 8.29 | 0.02 |
|  | Unknown | 1.26 | 0.30 | 5.26 | 0.76 |  | 1.28 | 0.24 | 6.71 | 0.77 |
| Mutation type | |  |  |  |  |  |  |  |  |  |
|  | L858R+T790M vs. 19Del+T790M | 1.04 | 0.66 | 1.61 | 0.88 |  |  |  |  |  |
| Metastasis before treatment | |  |  |  | 0.98 |  |  |  |  |  |
|  | No | 1.00 |  |  |  |  |  |  |  |  |
|  | Intrapulmonary metastasis | 1.17 | 0.43 | 3.18 | 0.77 |  |  |  |  |  |
|  | 1-3 Extrapulmonary metastasis | 1.04 | 0.41 | 2.66 | 0.94 |  |  |  |  |  |
|  | >3 Extrapulmonary metastasis | 1.07 | 0.41 | 2.81 | 0.89 |  |  |  |  |  |
| Progression pattern | |  |  |  | 0.10 |  |  |  |  |  |
|  | Asmptomatic | 1.00 |  |  |  |  |  |  |  |  |
|  | Brain metastasis | 1.17 | 0.40 | 7.57 | 0.47 |  |  |  |  |  |
|  | Systemic limited metastasis | 1.04 | 0.71 | 2.63 | 0.34 |  |  |  |  |  |
|  | Systemic multiple lesions | 1.07 | 0.56 | 3.36 | 0.48 |  |  |  |  |  |
|  | Unknown | 1.02 | 1.21 | 3.91 | 0.01 |  |  |  |  |  |
| Re-biopsy | |  |  |  |  |  |  |  |  |  |
|  | Yes vs. No | 0.84 | 0.54 | 1.31 | 0.45 |  |  |  |  |  |
| Resistance mechanism | |  |  |  | 0.97 |  |  |  |  |  |
|  | On-target mutation | 1.00 |  |  |  |  |  |  |  |  |
|  | Off-target mutation | 1.26 | 0.34 | 4.62 | 0.73 |  |  |  |  |  |
|  | SCLC transformation | 1.28 | 0.35 | 4.71 | 0.72 |  |  |  |  |  |
|  | Unknown | 1.06 | 0.54 | 2.06 | 0.88 |  |  |  |  |  |
| Post-OSI treatment | |  |  |  | 0.00 |  |  |  |  | 0.00 |
|  | Chemotherapy | 1.00 |  |  |  |  | 1.00 |  |  |  |
|  | Chemotherapy+ Anti-angiogenesis | 0.76 | 0.40 | 1.47 | 0.42 |  | 0.41 | 0.18 | 0.97 | 0.04 |
|  | Chemotherapy+ Osimertinib | 0.78 | 0.28 | 2.20 | 0.64 |  | 0.65 | 0.20 | 2.17 | 0.49 |
|  | Chemotherapy+ Immunotherapy | 0.84 | 0.33 | 2.14 | 0.71 |  | 0.63 | 0.24 | 1.68 | 0.36 |
|  | Osimertinib | 1.29 | 0.68 | 2.45 | 0.43 |  | 1.47 | 0.76 | 2.83 | 0.25 |
|  | Osimertinib+ Anti-angiogenesis | 0.39 | 0.19 | 0.83 | 0.01 |  | 0.20 | 0.08 | 0.49 | 0.00 |
|  | Best Support Care | 11.08 | 3.17 | 38.70 | 0.00 |  | 12.28 | 3.38 | 44.66 | 0.00 |
| LCT | |  |  |  |  |  |  |  |  |  |
|  | No vs. Yes | 0.78 | 0.45 | 1.37 | 0.39 |  |  |  |  |  |

**Supplementary Table 4. Univariate COX regression analysis by baseline characteristics for mOS**

| Variables | | Univariate analysis | | | |
| --- | --- | --- | --- | --- | --- |
|  |  | HR | 95%CI | | P |
| Gender | |  |  |  |  |
|  | Female vs. Male | 1.52 | 0.88 | 2.63 | 0.13 |
| Age | |  |  |  |  |
|  | ≥65 vs. <65 | 1.60 | 0.92 | 2.76 | 0.10 |
| PS | |  |  |  |  |
|  | ≥2 vs. 0-1 | 0.93 | 0.45 | 1.90 | 0.83 |
| Smoking history | |  |  |  |  |
|  | Yes vs. No | 0.73 | 0.37 | 1.43 | 0.36 |
| Pathology | |  |  |  |  |
|  | NSCLC-NOS VS. Adenocarcinoma | 1.84 | 0.82 | 4.16 | 0.14 |
| TNM stage | |  |  |  |  |
|  | IV vs. IIIB | 0.82 | 0.35 | 1.93 | 0.65 |
| Biopsy specimen | |  |  |  | 0.19 |
|  | Pulmonary tissue | 1.00 |  |  |  |
|  | Blood | 0.67 | 0.36 | 1.25 | 0.21 |
|  | Hydrothorax | 0.56 | 0.25 | 1.27 | 0.17 |
|  | Lymph node | 1.97 | 0.74 | 5.24 | 0.18 |
|  | Unknown | 0.00 | 0.00 | - | 0.98 |
| Mutation type | |  |  |  |  |
|  | L858R+T790M vs. 19Del+T790M | 1.37 | 0.79 | 2.37 | 0.26 |
| Metastasis before treatment | |  |  |  | 0.98 |
|  | No | 1.00 |  |  |  |
|  | Intrapulmonary metastasis | 1.17 | 0.43 | 3.18 | 0.77 |
|  | 1-3 Extrapulmonary metastasis | 1.04 | 0.41 | 2.66 | 0.94 |
|  | >3 Extrapulmonary metastasis | 1.07 | 0.41 | 2.81 | 0.89 |
| Progression pattern | |  |  |  | 0.40 |
|  | Asmptomatic | 1.00 |  |  |  |
|  | Brain metastasis | 1.17 | 0.43 | 3.18 | 0.77 |
|  | Systemic limited metastasis | 1.04 | 0.41 | 2.66 | 0.94 |
|  | Systemic multiple lesions | 1.07 | 0.41 | 2.81 | 0.89 |
|  | Unknown | 0.82 | 0.35 | 1.93 | 0.65 |
| Re-biopsy | |  |  |  |  |
|  | Yes vs. No | 0.66 | 0.37 | 1.16 | 0.40 |
| Resistance mechanism | |  |  |  | 0.66 |
|  | On-target mutation | 1.00 |  |  |  |
|  | Off-target mutation | 0.38 | 0.05 | 3.13 | 0.73 |
|  | SCLC transformation | 2.72 | 0.49 | 14.99 | 0.72 |
|  | Unknown | 2.27 | 0.83 | 6.22 | 0.88 |
| Post-OSI treatment | |  |  |  | 0.27 |
|  | Chemotherapy | 1.00 |  |  |  |
|  | Chemotherapy+ Anti-angiogenesis | 0.71 | 0.32 | 1.59 | 0.40 |
|  | Chemotherapy+ Osimertinib | 0.86 | 0.26 | 2.89 | 0.81 |
|  | Chemotherapy+ Immunotherapy | 1.23 | 0.37 | 4.14 | 0.73 |
|  | Osimertinib | 0.56 | 0.23 | 1.37 | 0.20 |
|  | Osimertinib+ Anti-angiogenesis | 0.40 | 0.17 | 0.95 | 0.04 |
|  | Best Support Care | 1.88 | 0.56 | 6.32 | 0.31 |
| LCT | |  |  |  |  |
|  | No vs. Yes | 0.73 | 0.36 | 1.46 | 0.38 |
