# Supplementary figures and images for: Osimertinib in combination with anti-angiogenesis therapy presents a promising option for osimertinib-resistant non-small cell lung cancer
Source: BMC Med. 2024 Apr 24;22:174. doi: 10.1186/s12916-024-03389-w (PMC11040894; doi:10.1186/s12916-024-03389-w)

## Slide 1
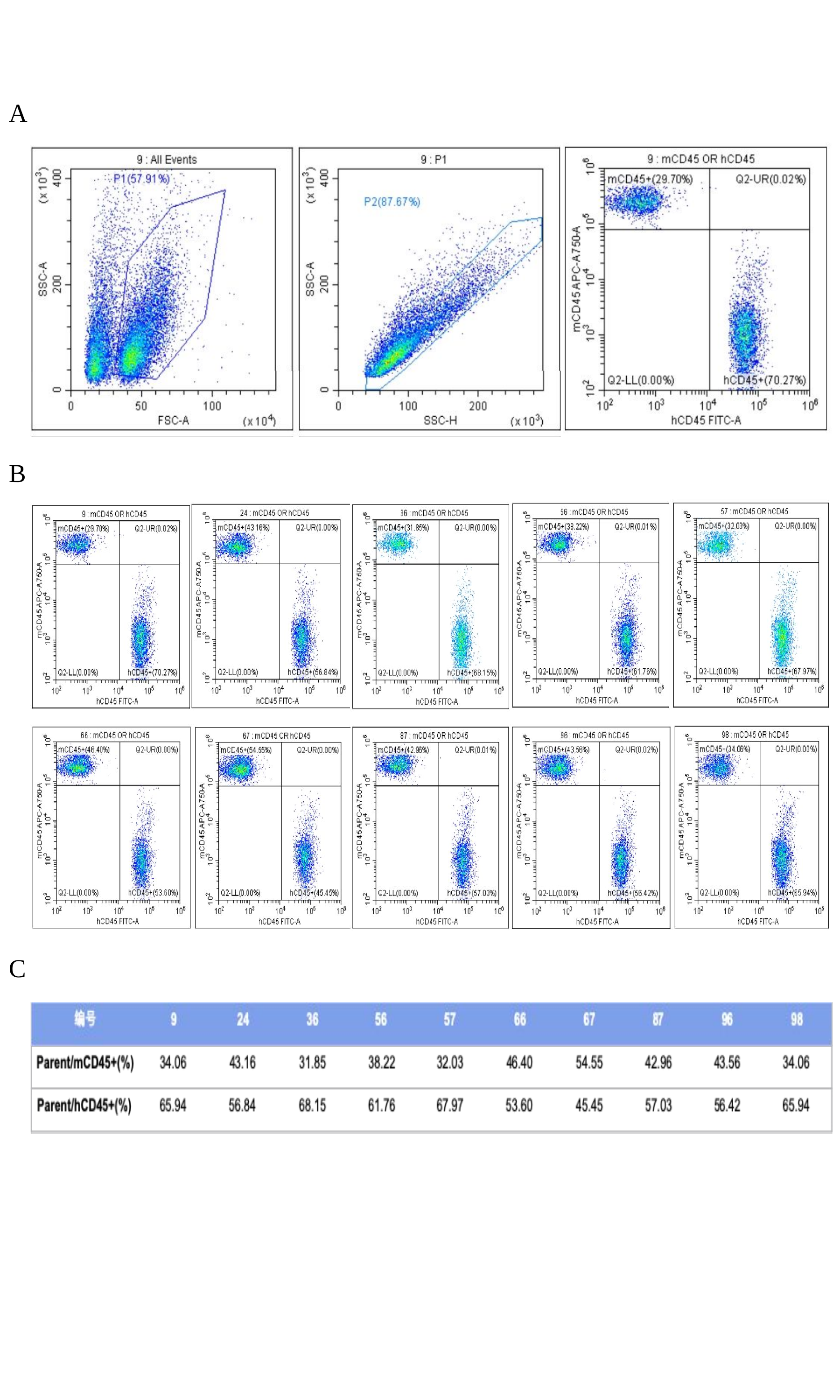

A
B
C

## Slide 2
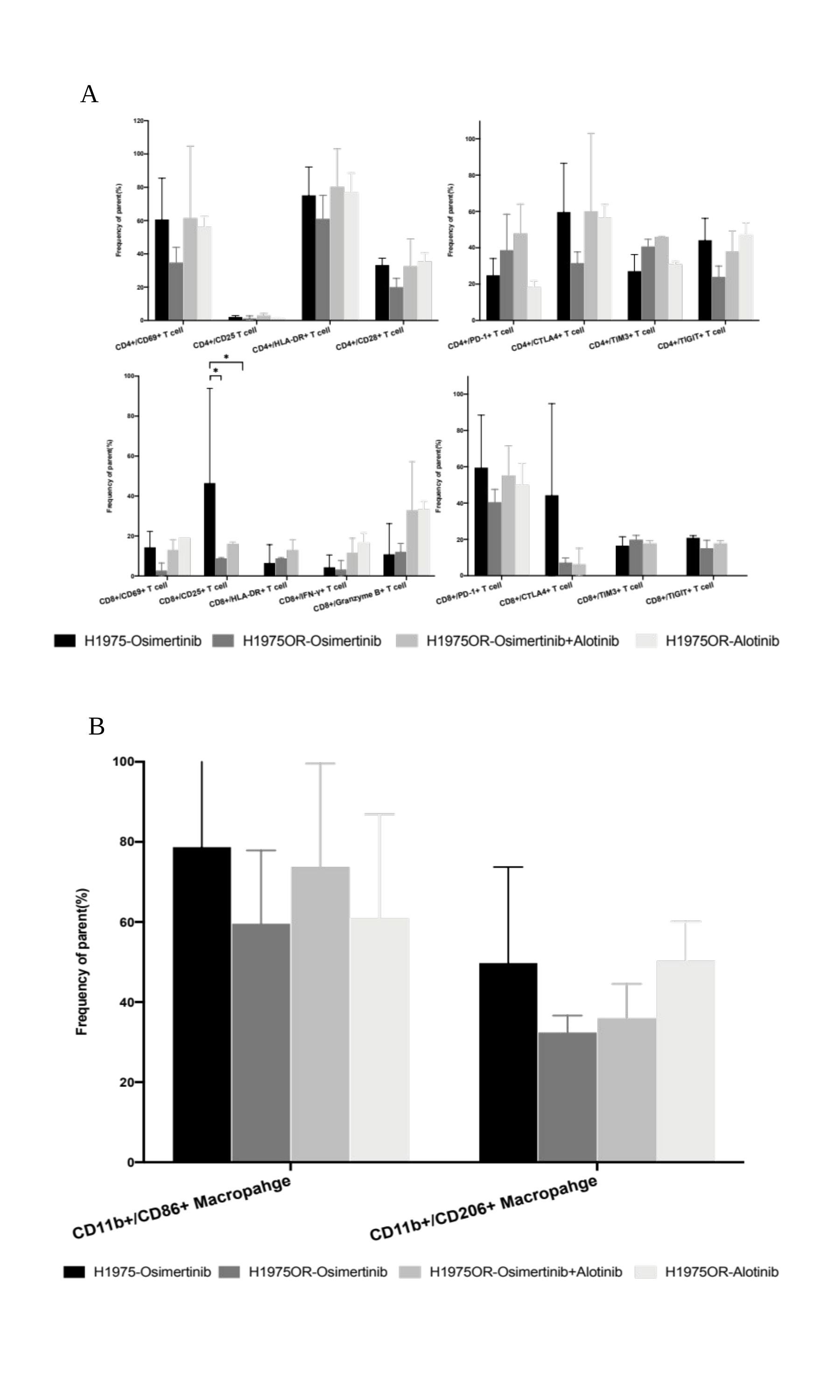

A
B

## Slide 3
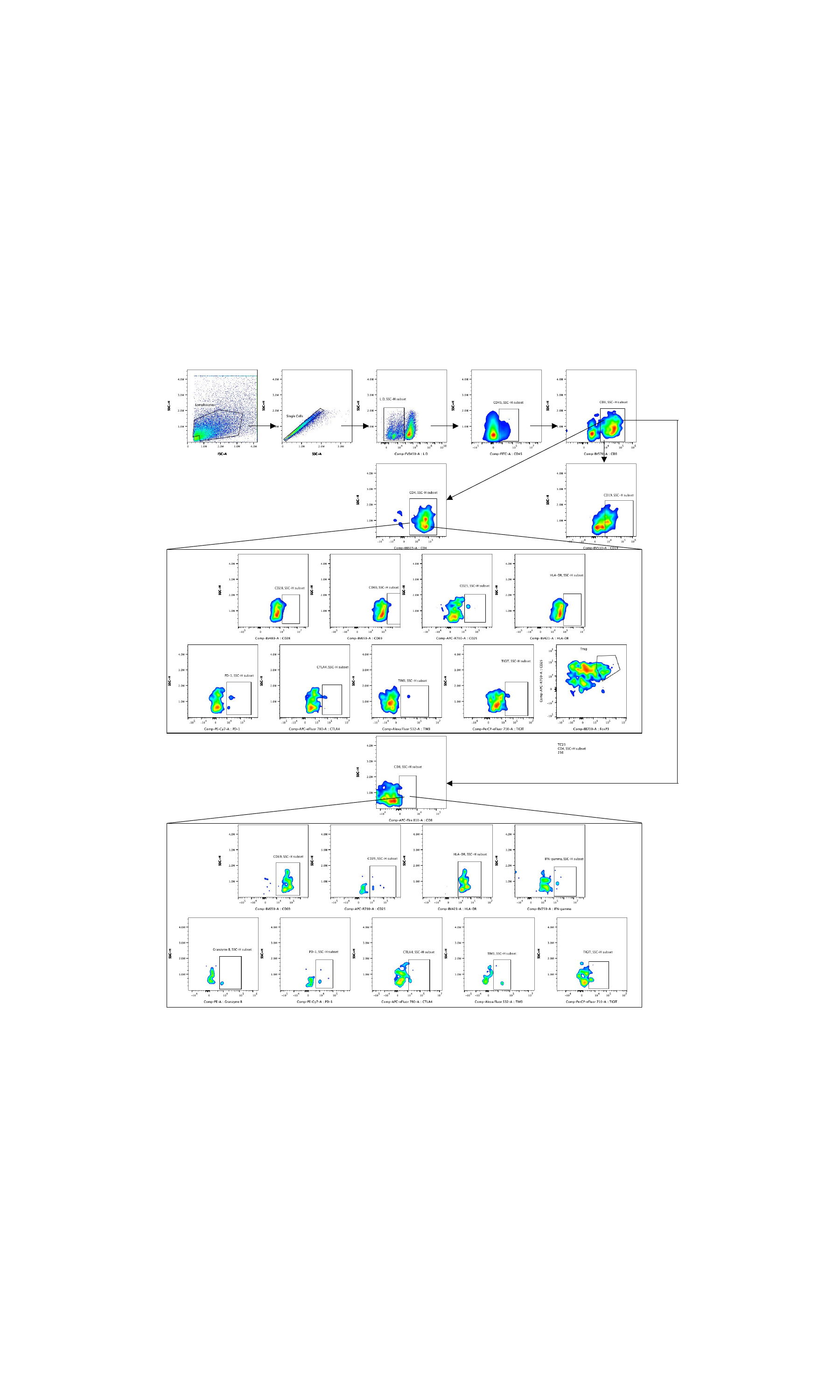

Supplement: Supplementary file 2 — Additional file 2: Fig S1. Evaluation of humanized mouse model. A. Gating strategy for detecting hCD45+ leukocytes in peripheral cells in mice; B. Flow cytometry results of each humanized mice constructed in our experiment; C. Proportion of hCD45+/mCD45+ in peripheral blood. Fig S2. Flow cytometry detection of infiltrating immune cells in peripheral blood. Comparative analysis of tumor-infiltrating T cells (A) and macrophages (B). Fig S3. Gating strategy of lymphocytes in tumor-infiltrating lymphocytes.Total leukocytes were gated with CD45+, in total lymphocytes were gated with CD3+. Then the CD4+ helper T cells, CD8+ cytotoxic T cells, and CD19+ B cells were then delineated in CD3+ lymphocytes, respectively. Next, CD4+ T cells were further divided into early activation (CD69+), middle activation (CD25+), late activation (HLA-DR+); exhausted (PD1+/CTLA-4+/TIM3+/TIGIT+), as well as immunosuppressive Treg (FOXP3+CD25+) T cells. Similarly, we also applied the above gating strategy in CD8+T cells. In addition, we checked IFN-γ and Granzyme B, which were related to killing functions. [file 12916_2024_3389_MOESM2_ESM.pptx]
